# The Truth Is Out There: Biological Features and Clinical Indications of Extracellular Vesicles from Human Perinatal Stem Cells

**DOI:** 10.3390/cells12192347

**Published:** 2023-09-25

**Authors:** Eleonora Russo, Giusi Alberti, Simona Corrao, Cesar V. Borlongan, Vitale Miceli, Pier Giulio Conaldi, Francesca Di Gaudio, Giampiero La Rocca

**Affiliations:** 1 Department of Biomedicine, Neurosciences and Advanced Diagnostics (BiND), University of Palermo, 90127 Palermo, Italy; eleonora.russo01@unipa.it (E.R.); giusi.alberti@unipa.it (G.A.); 2 Research Department, IRCCS ISMETT (Istituto Mediterraneo per i Trapianti e Terapie ad Alta Specializzazione), 90127 Palermo, Italy; corraosimona@libero.it (S.C.); vmiceli@ismett.edu (V.M.); pgconaldi@ismett.edu (P.G.C.); 3 Department of Neurosurgery and Brain Repair, Morsani College of Medicine, University of South Florida, Tampa, FL 33620, USA; cborlong@usf.edu; 4 Department of Health Promotion, Maternal-Infantile Care, Excellence Internal and Specialist Medicine “G. D’Alessandro” (PROMISE), University of Palermo, 90127 Palermo, Italy; francesca.digaudio@unipa.it

**Keywords:** mesenchymal stromal cells, human perinatal tissues, extracellular vesicles, cell-free therapy, Wharton’s jelly, umbilical cord, amniotic fluid, amniotic membrane, tissue repair

## Abstract

The potential of perinatal tissues to provide cellular populations to be used in different applications of regenerative medicine is well established. Recently, the efforts of researchers are being addressed regarding the evaluation of cell products (secreted molecules or extracellular vesicles, EVs) to be used as an alternative to cellular infusion. The data regarding the effective recapitulation of most perinatal cells’ properties by their secreted complement point in this direction. EVs secreted from perinatal cells exhibit key therapeutic effects such as tissue repair and regeneration, the suppression of inflammatory responses, immune system modulation, and a variety of other functions. Although the properties of EVs from perinatal derivatives and their significant potential for therapeutic success are amply recognized, several challenges still remain that need to be addressed. In the present review, we provide an up-to-date analysis of the most recent results in the field, which can be addressed in future research in order to overcome the challenges that are still present in the characterization and utilization of the secreted complement of perinatal cells and, in particular, mesenchymal stromal cells.

## 1. Introduction

In the last decade, significant advances have been made to fully assess the biology of mesenchymal stromal cells (MSCs) derived from perinatal tissues. The term “perinatal” defines birth-associated tissues and other fetal annexes (e.g., amnion, chorion, and the umbilical cord), obtained immediately after birth. To date, many applications of perinatal MSCs for clinical purposes have been studied with promising results, mainly due to their unique immune and differentiative properties [1]. Despite this, perinatal MSC-based therapy still presents problems related to different aspects, such as the difficulty of developing specific methods of isolation and characterization, as well as the identification of an optimal protocol for ex vivo expansion and the often observed efficacy post transplant [2,3]. Nevertheless, it has been suggested that the perinatal MSCs’ therapeutic efficacy may be fully recapitulated by their secretome or conditioned medium (e.g., soluble proteins, lipids, and extracellular vesicles) [4]. Thus, a new definition was introduced by Silini et al. namely “perinatal derivatives (PnD)” which includes all birth-associated tissues, the cells they are composed of, and all the biomolecules secreted [5]. Therapeutic approaches based on MSC secretomes may have different advantages over the use of transplanted MSCs, due to the minimal effect on immunogenicity, the higher yields of bioactive molecules, and easier application. Perinatal MSC secretomes seem to exhibit the same anti-inflammatory, immunomodulatory, and regenerative properties as parental MSCs [6,7,8], and appear to be effective in treating the side effects of ischemia–reperfusion injury [9,10]. Furthermore, the use of secretomes provides a suitable strategy to enhance MSCs’ therapeutic potential and standardize the production of MSC-derived products intended for clinical use [11]. Therefore, the utilization of secretomes could contribute to developing a novel, cell-free therapeutic approach.

The secretome is enriched with extracellular vesicles (EVs), secreted by cells through the development of multivesicular bodies or by cell membrane shedding [12,13]. They are classified into different types, based on their size, mechanism of biogenesis, functions, and tissue of origin, into: exosomes, microvesicles, ectosomes, and oncosomes [14]. Importantly, the cells (including the perinatal MSCs) are able to release various types of EVs [15]. EVs are present in almost every type of body fluid (e.g., plasma, urine, saliva, amniotic fluid, and others) and are therefore easily accessible. Once in the extracellular space, EVs facilitate cell-to-cell communication, between neighboring and distant cells, through transferring their molecular cargo (enriched in proteins, RNAs, and DNAs), both in physiological and pathological conditions [16,17,18]. The ISEV (International Society for Extracellular Vesicles) suggested using “extracellular vesicle” as the common term for all the vesicles released from cells to avert uncertainty within this complex field [19].

The increasing interest in the therapeutic benefits obtained by using the EVs released by parental MSCs in various diseases reinforces the hypothesis of the usefulness of MSC secretomes as a new therapeutic strategy.

## 2. Properties of Human Perinatal Tissue and Their Possible Therapeutic Application as Sources of EVs

**Figure 1:**
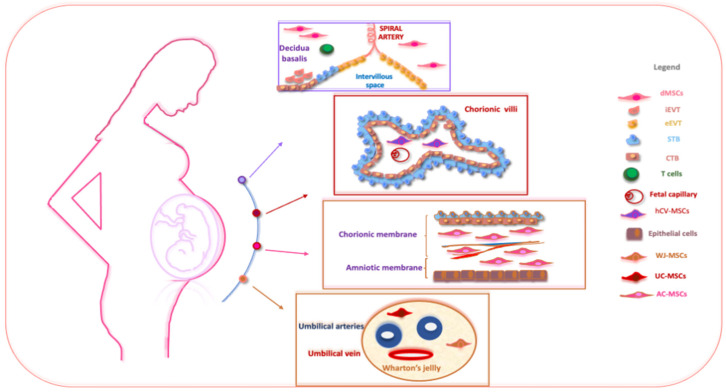
The schematic structure of the placenta and a description of perinatal MSCs. The placenta is an organ in which interactions between maternal and fetal cells, necessary for the development of the fetus, take place. Specifically, the placenta is made up of structures of fetal origin, such as the placental disc, the fetal membranes, divided into amniotic and chorionic membranes, and the umbilical cord, as well as a membrane of maternal origin called the decidua which originates from the endometrium. The chorionic villosity that forms the boundary between maternal and fetal blood during pregnancy represents the functional unit of the placenta. The different structures of the placenta are enriched by MSCs particularly useful in regenerative medicine and for the treatment of various pathologies. Abbreviations: dMSCs: decidua mesenchymal stromal cells; iEVT: inner extravillous trophoblast; eEVT: external extravillous trophoblast; STB: syncytiotrophoblast; CTB: cytotrophoblast; hCV-MSCs: human chorionic villi mesenchymal stromal cells; WJ-MSCs: Wharton’s jelly mesenchymal stromal cells; UC-MSCs: umbilical cord mesenchymal stromal cells; AC-MSCs: amnio-chorion mesenchymal stromal cells.

Perinatal tissues represent a plentiful source of promising cell types. Among the organs that may be used as a source of perinatal cells, the placenta is of key importance for its role in fetal nutrition and development, acting as a functional interface between the mother and fetus, either via the exchange of nutrients or via its immune and endocrine roles [20]. The formation of the placenta is the result of a series of processes that start in the moment of embryo implantation at the end of the first week of development. Then, the organ assists in the development of the embryo and then the fetus until delivery [21]. At delivery, the placenta features a diameter of 20–22 cm, with a thickness of up to 2.5 cm [22]. Structurally, the human placenta is a highly specialized essential organ with a distinction between the maternal-uterine part and the fetal part. Research related to perinatal MSCs began over a decade ago and has grown exponentially with the isolation and characterization of cells from different perinatal tissues [23,24,25] (Figure 1).

The placenta is usually discarded post-partum and easily obtained with a virtually limitless availability, free from ethical issues, and provides efficient MSC recovery without invasive procedures [26]. Different perinatal MSC populations are present and useful in regenerative medicine for the treatment of various pathologies, namely human decidua-MSCs (hD-MSCs), human syncytiotrophoblast and human cytotrophoblast (hSTB-MSCs and hCTB-MSCs), hAC-MSCs of the human amnio-chorionic membrane, as well as the MSCs present in the human umbilical cord (hUC-MSCs) and human amniotic fluid (hAF-MSCs).

MSCs are involved dynamically and actively in feto-maternal communication by releasing several molecules into the maternal circulation (including hormones and proteins) as well as EVs [27]. The therapeutic application of perinatal MSCs’ secretomes may present some advantages over the direct application of cells [28]. Indeed, it is now known that the therapeutic effects of perinatal cells are largely mediated by the secretion of EVs [11,29,30,31]. According to their size and biogenesis, EVs are distinguished into: (i) exosomes (30–150 nm), (ii) microvesicles (50 to 1000 nm, MV), and (iii) apoptotic bodies (1000–5000 nm) [32,33,34]. Of note, MSC-derived EVs were first isolated in 2010 in mice in a model of myocardial ischemia–reperfusion [35]. MSC-derived EVs are enriched with important bioactive molecules, such as mRNA and proteins, which regulate various biological processes. They express conventional markers of EVs (such as Hsp70, CD63, Flotillin-1, and TSG101) [22]. Nevertheless, they do not express costimulatory molecules, such as CD80 and CD86, thus conferring immune tolerance [36]. Proteomic analyses have allowed researchers to highlight the fact that perinatal-derived EVs are enriched in key proteins involved in several processes (e.g., immune, metabolic, and regenerative pathways) when compared to adult MSCs [37]. Currently, a few studies have attempted to characterize the miRNA profile of EVs isolated from perinatal MSCs [38,39], although some evidence is already available from experimental animal models [40,41]. One of these studies highlighted the presence of a few specific and highly expressed miRNAs, such as miR-145, miR-181c-5p, miR-Let-7e, and others [42]. Bulati et al. revealed that nine miRNAs found in human amnion MSC-derived EVs (hAMSC-derived EVs) were able to regulate the key proteins that control both T cell activation and monocyte differentiation [43]. Additionally, it has been reported that human umbilical cord MSC-derived EVs (hUCMSC-derived EVs) express high levels of miR16 and miR-Let-c which are involved in the regulation of T lymphocytes [44]. Perinatal MSC-derived EVs possess a lipid bilayer membrane also enriched with cholesterol, sphingomyelin, ceramide, and lipid raft proteins, which are involved in facilitating the trafficking and fusion of the membrane, bypassing any biological barrier [26]. Due to their specific properties, a number of studies have focused on EVs released by perinatal MSCs, highlighting their potential for cell-free therapy in clinical practice. Perinatal MSC-derived EVs are safer and have a longer shelf life than the MSCs themselves [45]. It has been observed that perinatal MSC-derived EVs have shown encouraging therapeutic effects in preclinical studies; five of these studies are reported in the www.ClinicalTrials.gov database accessed on 30 July 2023. One of these (NCT04213248) investigates the ability of umbilical cord-derived EVs to reduce dry eye symptoms in patients with chronic graft-versus-host disease (cGVHD). Another study (NCT04202770) evaluates the potential of liquid amniotic-derived EVs in the treatment of depression and neurodegenerative dementia, while another study (NCT04384445) evaluates their ability to suppress the activation of cytokines or intervene in other adverse events in COVID-19 patients with severe symptoms. In line with this, we herein summarize the current studies in the literature regarding cell-free therapeutic options by employing EVs derived from perinatal MSCs in the context of several pathological conditions.

### 2.1. Placenta-Derived EVs

As mentioned previously, perinatal tissues are obtained from term placentas and fetal annexes [5]. The maternal component of the placenta is the human decidua (hD), which is divided into three regions on the basis of relative position: the *decidua basalis* is the implantation site of the embryo and where the decidua interacts with the trophoblasts responsible for the forming of the basal plate; the *decidua capsularis*, enriched in decidual cells and some small vessels, is towards the uterus in contact with the chorion; and the *decidua parietalis* is an elongation of the decidua capsularis replacing the endometrium and merges with the capsularis, within the fourth month of gestation, in parallel with the reduction in uterine cavity width [46]. Depending on the spatial relation to the implanting embryo, hD-MSCs, derived from the maternal part of the placenta, may be isolated from both *decidua basalis* and *decidua parietalis* tissue. Generally, hD-MSCs have a shape similar to fibroblastic cells, the ability to adhere to plastic, and the presence of surface markers typical of MSCs, including HLA-ABC. Between the markers that are not expressed by hD-MSCs, studies have reported CD 40, CD80, CD83, CD-86, and HLA-DR [47]. Moreover, neither hematopoietic nor endothelial markers have been demonstrated in these cells [47]. From the differentiation point of view, it has been reported that hD-MSCs possess the competence to differentiate into the three classical mesenchymal cell lineages and, more importantly, also in tissue derived from all the three germ layers [48].

Placental tissue-derived EVs can be produced regardless of physiological or pathological condition; indeed, the biogenesis and release of EVs from the placenta may be regulated by changes in the cellular microenvironment, such as oxygen tension and glucose concentration [49,50]. Studies have demonstrated, for instance, that the release of EVs by placental MSCs is regulated by oxygen tension [50]. In normal pregnancy, placental EVs are increasingly released into the maternal circulation as the pregnancy progresses and are correlated with placental weight; however, in studies, in the first and second trimesters, an increase was observed, with a consistent decrease in the third trimester [51,52]. To confirm this, it was observed that the concentration of EVs was approximately 50-fold greater in maternal plasma in comparison with mean uterine artery blood flow [52]. Importantly, this concentration increases in pregnancies with complications, such as preeclampsia [53] and gestational diabetes [54] (Table 1). In line with this, EVs derived from placental MSCs used with different therapeutic effects are reported in Table 1.

Although the main roles of placental MSC-derived EVs have been studied in the maternal–fetal communication, it is interesting moreover to highlight their role in other conditions. For instance, it was shown that placental MSC-derived EVs alleviated oxidative stress and restored the biological activity in renal ischemia–reperfusion injury models by regulating mitochondrial structure and function [55]. Another work used high-resolution in situ imaging techniques to monitor the distribution of placenta MSC-derived EVs to kidney lesions in a mouse model of acute kidney injury (AKI). In detail, the authors observed the ability of placental MSC-EVs to stimulate tissue regeneration along with reducing the area of fibrosis [56]. Considering inflammatory bowel diseases, such as ulcerative colitis and Crohn’s disease, the potential of placenta MSC-derived EVs in their clinical therapy has recently been demonstrated [57]. In ulcerative colitis, placental-MSC EVs raise mucosal barrier defense, prompting decreasing intestinal inflammation and oxidative stress, which are the major pathophysiological factors in this disease [57]. Once released, MSC-derived EVs also exhibit regenerative capacity through the transfer of their molecular cargo. It has been observed that placental MSC-derived EVs mediate central nervous system (CNS) regeneration and functional repair following injuries by favoring the increase in endogenous NPCs’ proliferation and differentiation towards the neuronal lineage, as well as ameliorating motor and autonomic function in in vivo experiments [58]. Similarly, the neuroprotective properties of placental MSC-derived EVs were further shown in an experimental autoimmune encephalomyelitis model of multiple sclerosis [59]. Furthermore, it was observed that placental MSC-derived EVs exerted beneficial effects in Duchenne muscular dystrophy in vitro and in vivo studies, unlike the EVs derived from bone marrow MSCs (BM-MSCs) which had no ameliorative effects on the pathology [60]. Specifically, on the one hand, the molecular cargo of placental MSC-derived EVs stimulates muscle differentiation and, on the other hand, inhibit inflammation alongside fibrosis [58]. So far, cGVHD is a significant problem that is encountered with considerable incidence [63]. The ability of placental MSC-EVs to reduce the symptoms caused by cutaneous cGVHD has been reported, such as hyperpigmentations and ulcers caused by skin dryness [61]. Furthermore, placental MSC-EVs modulate inflammatory damage and intestinal dysbiosis, which are important in the development of the pathogenesis of myocardial infarction [62].

### 2.2. Chorion-Derived EVs

The fetal portion of the placenta is represented by the chorion, which underlines the decidua basalis grouping all the layers that enclose the embryo. It originates from the trophoblast, which unites with the extraembryonic mesoderm [64]. The so-called chorionic villi are finger-like projections of cytotrophoblast cells that characterize the surface of the chorionic membrane. The form of these placental villi varies with the type of implantation and placenta, and three weeks after fertilization, they are fully developed. The human chorionic plate (hCP) is made up of two different layers: the chorion and amnion, facing the intervillous space and the embryo, respectively. The hCP is externally lined by human syncytiotrophoblast (hSTB) and villous human cytotrophoblast (hCTB). Furthermore, where the cytotrophoblast meets and invades, the maternal decidua forms extravillous trophoblast (EVT). The EVT is located at the feto-maternal interface [62]. The villous trophoblast is characterized by two cell layers, that is, mononucleated cytotrophoblasts and the multinucleated hSTB. Interestingly, villous cytotrophoblast cells undergo morphological changes during pregnancy, with a cuboidal shape in early pregnancy which then gives way to more flattened cells at term [65]. The hSTB is a layer of highly differentiated cells that do not express the proteins of the major histocompatibility complex of class I or II (MHC-I and MCH-II) on the cell surface essential for its immunological protection [66].

The chorion per se does not generate blood vessels per se but represents the tissue in which the connecting vessels between the placental villi and the connective stalk (which later will give rise to the UC) are located [64]. Silini et al. have suggested nomenclatures to use for the MSCs present in them, that is, hCV-MSC for MSCs from the human chorionic villi and hCL-MSC for MSCs derived from the human chorion laeve [5].

During gestation, trophoblast cells perform key functions that depend on an increasing number of structural and functional changes occurring at their expense and mediated by the secretion of EVs [67]. For instance, a chromatographic study identified a specific marker of EVs released by hSTB, i.e., placental alkaline phosphatase (PLAP) [68], and it has been reported that PLAP is already present in the first trimester of normal pregnancy, but significantly increases towards the end of pregnancy [52]. Moreover, it has been demonstrated that hSTB continuously secretes EVs in the circulation of women with preeclampsia, and these EVs are particularly enriched in pro-inflammatory molecules, which can represent a danger signal for the detection of this pathology [69]. Moreover, hSTB-derived EVs contain increased levels of molecules with angiogenic effects that could be responsible for the alteration of maternal vascular responses [70]. Regarding hCTB-derived EVs, a few studies have explored their function. For example, a study showed that EVs can be released from the primary culture of hCTB cells in an oxygen tension-dependent manner [50]. Another study identified the enrichment of TNF-α marker in hCTB-derived EVs, with it able to increase decidual cell transcription and the secretion of NF-κB target cytokines (such as IL8, IL6, CCL2, and CXCL1), which could be a hallmark of a successful pregnancy [71].

Regarding MSCs derived from chorionic villi and chorion laeve, this distinction is only related to the different regions of origin, as these cell subpopulations do not differ from each other in their cellular characteristics and immunophenotypes [72]. Studies have demonstrated that hCV-MSCs express MSC markers similarly to other MSC lineages [73]. Specifically, they were positive for CD44, CD73, CD90, CD105, and HLA-ABC markers, but not for molecules such as HLA-DR, CD34, and CD19 [73]. Furthermore, they showed markers of embryonic stem cells, such as TRA-1-61 and TRA-1-80 [74]. The absence of the expression of immune markers renders them non-immunogenic. hCP-MSCs also express typical MSC markers [75]. Moreover, hCV-MSCs do not exhibit the expression of CD14, CD19, CD34, CD45, and HLA-DR surface antigens [75,76]; nevertheless, they feature HLA-G, which is responsible for their immunomodulatory properties [77]. Studies indicate that, compared to other perinatal stem cells, hCV-MSCs possess higher migration and proliferation properties [74,75]. hCL-MSCs are plastic adherent cells that reflect the criteria described by Parolini et al. [68].

Of note, MSC-derived EVs are also able to mediate many functions in pathological conditions: for example, MSC-derived EVs favored liver regeneration through antifibrotic and autophagic mechanisms in a hepatic failure model [78] and alleviated liver fibrosis induced by carbon tetrachloride (CCl_4_) in a mouse model [79] (Table 2). It was further remarked that chorionic plate MSC-derived EVs possess the ability to mediate angiogenic effects in in vitro studies and are also able to strengthen angiogenesis in in vivo experiments [80] (Table 2). Considering the EVs released from EVTs, it has been observed that in conditions of low oxygen tension, EVT-derived EVs have the ability to increase the production of TNFα by human umbilical vein endothelial cells (HUVECs), which results in a decrease in their migratory capacity [81]. Lastly, EVs from hSTBs, hCTBs, EVTs, and placental promote the migration of vascular cells necessary for angiogenesis during pregnancy [50].

### 2.3. Chorionic- and Amniotic Membrane-Derived EVs

The human amniotic membrane (hAM) lines the amniotic cavity containing the amniotic fluid (AF). Due to the extension and cellular heterogeneity of the amniotic membrane, a subdivision in different zones has been proposed: the peripheral part of the membrane that lines the *chorion laeve* has been indicated as the human reflex amniotic membrane (hRAM). Instead, the portion of the membrane that lines the chorionic plate has been named the human placental amniotic membrane (hPAM). The dramatic growth of the amniotic cavity, starting at the fourth week of development, increases the contact between the hAM and the chorion, leading to the formation of the human amnio-chorionic membrane (hACM). Overall, the hACM constitutes the union between the fetal membranes with the chorionic plate [82]. Specific abbreviations have been suggested according to the different cell subpopulations, namely: (i) human amniotic epithelial cells (hAECs), (ii) human amniotic mesenchymal stromal cells (hAMSCs), (iii) human chorionic mesenchymal stromal cells (hCMSCs), and (iv) human chorionic trophoblastic cells (hCTCs) [25]. Moreover, some minimal criteria have been established for defining hAMSCs and hCMSCs, which include: (i) the ability to adhere to plastic in in vitro conditions; (ii) the expression of MSC markers such as CD90, CD73, and CD105 and a lack of hematopoietic molecules such as CD45, CD34, CD14, and HLA-DR; and (iii) the ability to differentiate into one or more lineages, including osteogenic, adipogenic, and/or chondrogenic [70]. The hAMSCs exhibit a fibroblast-like cellular shape and retain their morphology until five passages in vitro [83]. Furthermore, immunophenotype studies have confirmed that hAMSCs express CD29, CD44, CD73, CD90, CD105, CD166, CK18, HCAM-1, and human leukocyte antigen HLA-ABC while being negative for markers associated with pluripotency [70,83,84,85]. Regarding hCM-derived MSCs, they present a fibroblast-like morphology, plastic adherence capacity, and differentiation potential consistent with the criteria reported by Parolini et al. [70]. hCM-derived MSCs express the most commonly reported markers on their surface, such as CD13, CD29, CD44, and others, but they are absent of the CD3, CD14, CD34, CD45, and CD31 markers [86].

Thence, hAM- and AM-derived cells, at the end of pregnancy, represent a highly abundant and accessible tissue that could be useful for diverse therapeutic applications [87]. hAECs have been the focus of extensive research over the past decades for their therapeutic potential in various pathological conditions, especially considering their paracrine effects as well as the production of EVs (hAE-derived EVs) [88]. It has been observed that in a chemotherapy-induced premature ovarian failure (POF) mouse model, hAEC-derived EVs restored ovarian function through inhibition of granulosa cell apoptosis and protecting the ovarian vasculature from damage via the transfer of microRNAs [89] (Table 3). Considering this aspect, in Table 3, we summarize studies that exploit the advantages gained by using EVs derived from fetal membrane MSCs.

In immortalized human proximal tubular cells, hAEC-derived EVs ameliorated renal tissue damage, preventing apoptosis, as well as capillary rarefaction together with immunomodulation activity [90]. Similarly, other studies have reported the positive biological effects mediated by hAEC-derived EVs in diabetic [91] and non-diabetic wound healing conditions [92]. Of note, diabetic wounds represent a serious complication of diabetes mellitus, for which the optimal therapeutic tools are still unknown. It was shown in an in vitro study that hAEC-derived EVs favor the proliferation and migration of human fibroblasts (HFBs) and human umbilical vein endothelial cells (HUVECs) and stimulate collagen deposition, angiogenesis, and the wound-healing process in in vivo experiments [91], as well as accelerating wound healing in a normal physiological process through their protein and miRNA content [90]. Several in vitro studies have examined the immunomodulatory effect of hAEC-derived EVs. Treatment of a mouse model of CCL4-induced hepatic fibrosis and BM-derived macrophages with hAEC-derived EVs led to an increase in the polarization of the macrophages towards the M2 phenotype and, consequently, a significant reduction in liver fibrosis and macrophage infiltration, respectively [93]. The immunomodulatory properties of hAEC-derived EVs were also assessed in different immune cell populations in comparison to EVs obtained from human lung fibroblasts (hLF-EVs) in lung fibrosis conditions by Tan and coworkers [98]. It was observed that hAEC-derived EVs are able to directly reduce idiopathic pulmonary fibrosis and bleomycin-induced lung injury by polarizing and increasing macrophage phagocytosis and by reducing neutrophil myeloperoxidase, as well as by suppressing T cell proliferation directly via activation of the PI3K–AKT–mTOR pathway [92]. The biological capacity of hAEC-derived EVs was further explored by Sheller and colleagues in an oxidative stress situation induced by cigarette smoke during pregnancy [94]. It is therefore evident that hAEC-derived EVs have anti-inflammatory properties given their enrichment in factors that participate in immunomodulation, such as P38, PI3K-Akt, and other proteins. Taken together, these results demonstrate that hAEC-derived EVs have important repair potential in the treatment of various diseases, imitating the effects of cell transplantation and avoiding the related shortcomings.

hAMSCs-derived EVs’ functional and molecular characterization remain fragmentary, thus delaying their translation into clinical practice. Recently, hAMSC-derived EVs were observed to mediate the healing of inflamed and diseased joints and tendons due to the fact that they possess teno/chondroprotective characteristics, as well as the ability to induce the polarization of M2 macrophages and the inhibition of inflammatory T cells, with consequent promotion of Treg [95]. In this regard, miRNAs have been identified in hAMSC-EVs with a cartilage protection role, namely miR-146a-5p and miR-24-3p and other less-expressed miRNAs [95]. Additionally, the use of hAMSC-derived EVs allowed for a reduction in fibrosis and extracellular matrix deposition, as well as the inhibition of the expression of fibrotic genes in HSCs under hypoxic conditions rather than normoxic conditions [96].

Recently, EVs derived from the conditioned medium of hCMSCs (hCMSC-EVs) have been isolated and characterized, demonstrating that EVs and their contents can be taken up by different types of mesenchymal cells (i.e., synovial fibroblasts, osteoblasts, and periosteum-derived MSCs) during the osteoarthritic process, suggesting their perspective role in the treatment of disease [97].

### 2.4. Amniotic Fluid-Derived EVs

At around the seventh/eighth day of embryonic development, between the membrane that directly covers the embryo (amnios, formed by amnioblasts) and the embryo itself, a small space is formed, which will give rise to the amniotic cavity filled with AF [99]. Importantly, the AF is mainly composed of water (99%) and different nutrients and chemicals that are constantly and rapidly exchanged between the fetus and the mother during gestation. In addition, AF allows for fetal movement and growth within the uterus [100]. AF represents another rich source of perinatal MSCs, with minimal ethical concerns associated with its isolation. In general, they are collected safely during second-trimester routine amniocentesis, third-trimester amnioreduction, or at the end of gestation, showing only minimal replicative senescence, which makes them a particularly attractive source of stromal cells. AF includes a heterogeneous population of cells with phenotype and differentiation potential variable according to the gestational age [101] and different donors [102]. So far, AF can be collected through a safe procedure (amniocentesis), a technique employed for the prenatal diagnosis of chromosomal abnormalities and fetal infections. In the literature, several studies are present that highlight the presence of different populations of human amniotic fluid-derived mesenchymal stromal cells (hAF-MSCs), with a broad differentiation potential toward mesenchymal lineages (e.g., adipogenic, osteogenic, chondrogenic, and myogenic), as well as ectodermal (neurogenic) lineages, and they possess the ability to adhere to plastic [103]. In addition, hAF-MSCs are cells with a spindle-shape, are highly proliferative, and are able to form colonies, expressing surface markers that overlap with some MSC markers (such as CD73 and CD90), but do not express hematopoietic and endothelial markers (CD45, CD34, and CD31). It has also been assessed that hAF-MSCs express Oct-4, especially at the first passages of an in vitro culture compared to later passages [104]. Moreover, hAF-MSCs possess SSEA4, TRA-1-60, and TRA-1-81 markers [105]. Importantly, AF can be employed as a source of cells with higher potency and with the ability to differentiate into cells of all three embryonic germ layers without forming tumors, identified as amniotic fluid stromal cells (AFSCs) [106]. hAF-MSCs present many properties that could resemble human embryonic stem cells (ESCs), with them appearing to be safer and more pluripotent than stem cells derived from BM [107,108]. Consequently, hAF-MSCs represent a new class of stromal cells with properties of plasticity intermediate between embryogenic and adult stem cell types [107,108]. Therefore, hAF-MSCs could be cultured and modulated ex vivo under specific growth conditions and used for stem cell therapy.

In relation to the therapeutic utility of hAF-MSCs, various beneficial effects are known to be mediated by secreted EVs (hAFMSC-derived EVs). Balbi and coworkers showed that hAFMSC-derived EVs act as functional mediators of proliferative, antiapoptotic, immunomodulatory, proangiogenic, and anti-inflammatory processes, which are key properties for regenerative therapy. In particular, they have shown that hAFMSC-derived EVs modulate the expression of inflammatory (Il-1α, Il-6, and Il-4) and pro-resolving (Il-10) cytokines, as well as blocking the infiltration of immunoglobulins into inflamed muscle tissue [109]. In Table 4, we explored the potential benefit obtained with the use of EVs derived from hAF-MSCs.

As shown in Table 4, hAFMSC-derived EVs are capable of delivering neuroprotection directly by favoring anti-apoptotic and pro-survival pathways in an oxygen and glucose deprivation stroke model (ischemia–reperfusion model) [110]. Several miRNAs are also able to confer neuroprotection in a stroke model, such as miR-146a-5p, miR154-5p, miR22-3p, and others [106]. Interestingly, hAFMSC-derived EVs present immunoregulatory properties that have been demonstrated to vary according to gestational age in an animal model of osteoarthritis [111]. In this work, hAFMSC-derived EVs were able to counteract cartilage damage by modulating macrophage polarization (M2 phenotype), influencing the polarization of Treg and reducing the maturation of memory B cells, as well as increasing pain tolerance [111]. About that, Beretti et al. found that hAFMSC-derived EVs from conditioned medium (CM) act on the reduction in the proliferation of lymphocytes, in particular on the subpopulation of T helper cells, while the hAFMSC-CM deprived of EVs was shown to promote apoptosis. Moreover, through proteomic analyses, they found differential EV cargo in hAFMSC-CM compared to AF and slightly higher content of proteins involved in cell growth, such as in the HGF and TGFβ 1 and 2 hAFMSC-CM samples [117]. In ischemic myocardial infarction, hAFMSC-derived EVs’ administration in mice results in a prolonged cardio-active effect with improvement in cardiac function up to 1 month from treatment [112,113]. Likewise, the administration once a week of hAFMSC-EVs improved wound healing in a preclinical rat model of cutaneous injury [114].

It is known that hAFMSC-derived EVs provide information specific to normal and abnormal parturition. In this regard, a proteomic profile analysis, with the enrichment of key inflammatory markers, allowed us to distinguish hAFMSC-derived EVs isolated from patients who had spontaneous preterm birth or premature rupture of membranes compared with those who delivered at term [118]. Furthermore, it was observed that there was differential expression of antiangiogenic factors in normal and preeclamptic hAFMSC-derived EVs, both at the surface and cargo levels, reflecting the general hypoxic and antiangiogenic status of preeclampsia conditions [119]. Considering pediatric diseases and malformations, hAFMSC-derived EVs may represent important therapeutic tools. For instance, in necrotizing enterocolitis (NEC), a devastating intestinal disease primarily affecting preterm neonates, hAFMSC-derived EVs may act as intercellular messengers able to attenuate intestinal injury in a Wnt-dependent manner [115].

Interestingly, it has been reported that other features of hAFMSC-derived EVs may reside in their anti-apoptotic, pro-angiogenic, and immune-modulatory activities, prospectively of interest in anticancer therapy. For example, hAFMSC-derived EVs were shown to inhibit apoptosis and support the survival of damaged granulosa cells, thus preventing ovarian follicles from atresia in mice following chemotherapy [116].

### 2.5. Umbilical Cord-Derived EVs

The hUC connects the embryo, and then the fetus, to the placenta, allowing the exchange of nutrients and gasses during gestation. The UC is formed when the body stalk (including the allantois), umbilical blood vessels, and allantoid duct along with the umbilical coelom are surrounded by diffuse amnion. The extraembryonic mesoderm of all the ducts in the UC merges together, giving rise to a mucoid connective tissue called “Wharton’s jelly” (human umbilical cord Wharton’s jelly, hUC-WJ) described for the first time by Thomas Wharton in 1656 [120]. This particular tissue features a population of cells resembling fibroblasts which are enclosed in an abundant extracellular matrix and largely formed by amorphous substances (proteoglycans) with lesser fibrillar molecules (collagen, elastic, and reticular fibers). The external layer of the UC is lined by a specialized epithelium (mono- to pluristratified), named the periderm, of ectodermal origin. The UC contains three vessels, one vein and two arteries, which mediate the exchange of oxygenated/deoxygenated blood and nutrients between the placenta and the fetus [121]. At term gestation, the UC is approximately 50 cm long and 2 cm in diameter [117,118]. Considering the MSCs derived from the UC, the nomenclature accepted is hUC-AMSCs for amniotic MSCs derived from the hUC, hUC-WJ-MSCs for MSCs isolated from hUC-WJ, hUC-saWJ-MSCs for MSCs obtained by human umbilical cord sub-amnion Wharton’s jelly, and hUC-iWJ-MSCs for MSCs from a lineage of human umbilical cord intermediate Wharton’s jelly [122,123,124]. Finally, the nomenclature for MSCs isolated from AF is hAF-MSCs, whereas for MSCs obtained by human basal and parietal decidua, it is hBD-MSCs and hPD-MSCs, respectively [5].

The cells can be isolated in both the UC blood UCB and UC tissue [5]. hUC-MSCs are fibroblast-like and plastic adherent cells, expressing CD13, CD29, CD44, CD73, CD90, and CD105 markers but lacking HLA-DR and other markers. Furthermore, they could give rise to cells belonging to all three germ layers [125]. It is generally acknowledged that for regenerative medicine applications, hUC-MSCs may represent an excellent alternative to BM-MSCs. Comparative studies have shown that the former is less immunogenic, features higher proliferation rates, and has greater anti-inflammatory properties [126,127]. Currently, there is a consensus that MSCs isolated from Wharton’s jelly display characteristics of other perinatal MSCs [68]. Interestingly, hUC-WJ-MSC can have two different morphologies: cells with a flat cell body or slender fibroblast-like cells [128]. We and others around the world have extensively studied the isolation, characterization, and differentiation potential of hUC-MSCs. They express CD10, CD13, CD29, CD44, CD54, CD73, CD90, CD105, Stro-1, MHC class I (classical HLA-A, -B, and -C and non-classical HLA-G, -E, and -F) but are negative for the hematopoietic and endothelial markers CD14, CD19, CD31, CD34, CD38, CD45 CD66b, CD80, CD86, CD106, CD133, and HLA-DR [129,130,131,132]. In the literature, it has also been reported that hUC-WJ-MSCs express TRA-1-60, TRA-1-81, SSEA-1, and SSEA-4, even if their expression is questionable due to the discordant and uncertain results. As pluripotent stem cell markers, hUC-WJ-MSCs feature NANOG, Rex-1, and Sox-2 [133,134,135,136]. Like other populations of perinatal MSCs, UC-MSCs can differentiate into cells belonging to all germ layer derivatives [137,138]. Moreover, the hUC features the expression of immunosuppressive factors, such as TGF-β2, suggesting their role in immune modulation [138]. When compared to adult-derived MSCs, hUC-MSCs have a higher production rate of EVs as part of their complex secretome and their biological roles [139]. Importantly, the results of a cytokine profile analysis revealed that hUCMSC-derived EVs contained GM-CSF, IL-15, IL-6, IL-8, TNF-α, IL-1β, IL-2, and IL-10 [140]. Li et al. showed that hUCMSC-derived EVs ameliorate the CCl_4_-induced liver fibrosis in vivo model by mediating hepatic protection and inhibiting the detrimental epithelial-to-mesenchymal transition of hepatocytes [141]. As described in Table 5, umbilical cord UCMSC-derived EVs have been tested for different therapeutic applications.

Similarly, hUCMSC-derived EVs have provided evidence of their ability to accelerate the functional and morphological recovery of cisplatin-induced acute kidney injury by promoting proliferation and damage repair once incorporated into damaged epithelium, a result that has been observed both in vitro and in vivo [142]. Interestingly, IV-administered hWJMSC-derived EVs preserved kidney function and decreased serum levels of the AKI marker neutrophil gelatinase-associated lipocalin in a unilateral kidney ischemia model [143], and hUBCMSC-derived EVs are instead suggested for the treatment of liver fibrosis [144]. Furthermore, hUCBMSC-derived EVs improve dextran sulfate sodium-induced inflammatory bowel disease through the modulation of IL-7 expression in macrophages [145], whereas by inhibiting inflammatory cell migration into the eye, they ameliorate the autoimmune disease uveoretinitis [146]. As therapeutic tools, hUCMSCs-derived EVs have also been explored for myocardial ischemia–reperfusion injury, since it was observed that they protected myocardial cells from apoptosis alongside promoting angiogenesis and cell proliferation [147]. It has been demonstrated that hUCMSC-EVs are enriched in miR-19, with them able to protect cardiomyocytes against myocardial infarction [148]. Additionally, hUCMSC-EVs played an active role in repairing mifepristone-injured human endometrial stromal cells [149]. In atopic dermatitis [150] and wound healing [141], hUCMSC-derived EVs transferred many factors to recipient cells, such as VEGF, MCP-1, IL-6, and IL-8, involved in inflammation processes. In vitro and in vivo studies confirmed that hUCMSC-derived EVs can interact directly with rat primary microglia cells in a model of traumatic spinal cord injury (SCI) through anti-inflammatory effects (e.g., reducing the expression of IL-1β and IL-6) and mediating a reduction in scarring activities, mimicking parental MSCs in the early phase of secondary injury [152]. Importantly, in an in vitro study of hypoxic–ischemic injury in the perinatal phase, let-7-5p miRs in hWJMSC-derived EVs were found to confer neuroprotection and stimulate neuroregeneration while limiting the severity of injury [153]. Furthermore, it has also been shown that engineered hUCMSC-EVs, featuring CD73 (ecto-5′-nucleotidase) overexpression, ameliorated inflammation in a mouse model of SCI [159].

In lung cancer cell lines, hUCMSC-derived EVs are capable of promoting migration and invasion by transferring miR-410 [154]. Instead, in pancreatic ductal adenocarcinoma and in breast cancer, the hUCMSC-derived EVs inhibit cell proliferation and invasion, as well as favoring cell cycle arrest and apoptosis [155,156]. Contradictory results obtained by Zhou and colleagues showed that hUBMSC-derived EVs promoted the invasion and migration of breast cancer cells and promoted the induction of EMT through the activation of the ERK pathway [160]. Likewise, Zhao et al. found that hUCMSC-derived EVs are also able to promote EMT transition in lung cancer, although inhibition of EMT is achieved once the TGF-β pathway in MSCs is blocked [161]. Of note, hWJMSC-derived EVs promoted anti-proliferative and pro-apoptotic effects in bladder carcinoma [157]. Moreover, it has been found that hUCMSC-derived EVs are enriched in miR-124, which acts as a tumor suppressor in various cancers [162].

In perinatal brain injury treatment, WJ-MSCs have been shown to reduce neuroinflammation and induce neuro-regeneration [163]. The effects of hWJMSC-derived EVs may reside either in reducing microglial inflammation, as observed by Thomi and coworkers, or, in particular, by interfering with the TLR4/CD14 signaling cascade. The authors concluded that this may lead to a reduction in the transcriptional levels of inflammation-related genes [29]. Furthermore, hWJMSC-derived EVs mediated neuroprotection against amyloid beta oligomer (AβO)-induced neuronal oxidative stress and synapse damage in an in vitro model of Alzheimer’s disease (AD) and for other neurodegenerative disorders [164]. The hWJMSC-derived EVs containing miR-30 improved renal recovery in an ischemia–reperfusion injury model [165]. Furthermore, it has also been shown that EVs derived from human umbilical vein endothelial cells (HUVEC-derived EVs) can be used in regenerative medicine applications. In particular, these cells can act positively on neural stem cells (NSCs) both in terms of proliferation and stemness, thus displaying, almost in vitro, the potential to expand the NSC pool during brain regeneration [166]. For instance, HUVEC-derived EVs may reduce oxidative stress by inhibiting mitochondrial RNA-processing endoribonuclease in neurons via the induction of miR-206/miR-1-3p levels [167]. Moreover, the UCB represents a source of MSCs with key immunomodulatory properties during the maintenance of pregnancy, and not only that, but they also possess capabilities to release EVs (UCBMSC-derived EVs) with important immunosuppressive functions [158].

## 3. New Technologies for the Application of EVs

Perinatal MSCs are emerging as a strong candidate for a new source of MSCs that could be suitable to replace adult MSCs as they can be procured by noninvasive procedures and there are no ethical controversies surrounding them. It is beyond doubt that most of the perinatal sources of stem cells that we have reviewed here present key improvements over adult MSCs in terms of availability, proliferation rate, efficacy, and safety for both donor and recipient [168,169,170,171]. However, the poor efficacy of cell therapy is one of the factors that considerably limits its use. In the past decade, evidence has accumulated showing that EVs can display numerous biological properties that vary according to their parental cells. The publications cited in the previous sections outline an extensive and growing list of potential applications of EVs derived from perinatal tissue in the treatment of different diseases. Recent research has shown the beneficial effects of EVs released from perinatal MSCs per se but they can also be bioengineered to generate an EV product with enhanced or altered therapeutic properties by genetic engineering or priming, post-release modifications, and other methods [11,172]. Therefore, these approaches require maintaining the physicochemical stability and functional stability of the engineered vesicles and maintaining the safety of the final product. To date, biomaterials of different kinds, such as hydrogels or scaffolds, are being widely employed in order to deliver EVs locally and for tissue engineering, thereby prolonging the retention of EVs at target sites and ameliorating their therapeutic efficacy delivery [173,174]. It has been shown that UCMSCs yielded four times as many EVs per cell than MSCs from BM or adipose tissue did in scalable three-dimensional (3D) microcarrier-based cultures, thus demonstrating that EVs derived from the 3D culture of UCMSCs exhibited a superior curative effect in the treatment of cartilage defects compared to the 2D culture [175]. Another study developed a collagen scaffold laden with hUCMSC-derived EVs in a rat endometrium-damage model. The authors showed that the in vivo transplantation of the scaffold packaged with EVs enhanced endometrial regeneration and collagen remodeling, as well as fertility restoration. Of note, the in vitro and in vivo experiments carried out have highlighted the close relationship established between the scaffold potency containing the EVs and the polarization of the M2 macrophages, responsible for this miRNA enriched in EVs [176]. Yang et al. reported the effectiveness of the delivery of hUCMSC-derived EVs in pluronic F-127 hydrogel for the treatment of chronic diabetic wounds. In fact, the wound healing process was faster with overall better epithelial regeneration when a composite of exosomes in a hydrogel was used with respect to the exosomes alone or the hydrogel alone [177]. Moreover, hUCMSC-EVs encapsulated in hydrogel scaffolds provide myocardial regeneration [178]. Finally, this review provides a concise analysis of current perinatal MSC-derived EV-based clinical applications.

## 4. Future Perspectives

Overall, EVs secreted from perinatal MSCs exhibit key therapeutic effects such as tissue repair and regeneration, the suppression of inflammatory responses, immune system modulation, and a variety of other functions. Although the properties of MSCs-derived perinatal EVs and their significant potential for therapeutic success are recognized, several challenges still remain that need to be addressed. First of all, from a practical point of view, the standard methodologies for the isolation and large-scale production of EVs, as well as their characterization and administration, require standardized protocols to be applied as effective, safe, and potent cell-free therapies. Overall, the knowledge in the research underlining the therapeutic mechanism of action of perinatal MSC-derived EVs, together with the development of preclinical models to test their therapeutic efficacy could contribute to new findings on the applicability of perinatal MSC-derived EVs necessary to achieve translation and successful clinical implementation.

## Figures and Tables

**Table 1:** Therapeutic advantages of EVs released by placental MSCs.

EVs’ Source	Disease Model	In Vitro and/or In Vivo Experiments	Molecular Mechanism Proposed	Pathophysiological Effects	References
Placental MSC-derived EVs	Preeclampsia	In vitro	Increase in migration, placenta development, and angiogenesis pathways.	+ The early detection of women at risk + Preventative therapy + Improves pregnancy outcomes for both mother and baby	[53]
Placental MSC-derived EVs	Gestational diabetes	In vitro	Increase in PI3K/Akt, Wnt, insulin, and mTOR signaling pathways.	+ Insulin resistance − Oxidative stress − Inflammation	[54]
Placental MSC-derived EVs	Acute kidney injury (AKI)	In vivo	Activated Kelch-like ECH-associated protein 1 (Keap1)/nuclear factor E2-related factor (Nrf2) signaling.	+ Mitochondrial antioxidant defense − Inflammation − Apoptosis	[55]
Placental MSC-derived EVs	AKT	In vivo	Increase in Sox9+ expression and decrease in α-SMA, fibronectin, collagen I, and TGF-β1.	+ Proliferation + Regeneration − Apoptosis − Fibrosis d28	[56]
Placental MSC-derived EVs	Ulcerative colitis	In vitro and vivo	Decrease in pro-inflammatory cytokines (such as TNF-α, IL-1β, IFN-γ, and IL-6) and the upregulation of anti-inflammatory cytokines, such as IL-10 and TGF-β.	+ Mucosal healing − Inflammation − Oxidative stress	[57]
Placental MSC-derived EVs	Spinal cord injury	In vitro and vivo	Increase in SOX2^+^GFAP^+^ and PAX6^+^Nestin^+^ and the activation of MEK/ERK/CREB pathway signaling.	+ Neurogenesis + Hind limb locomotor recovery + Bladder dysfunction	[58]
Placental MSC-derived EVs	Multiple sclerosis	In vitro and vivo	Modulation of T regulatory populations, i.e., shifts of Th1 to Th2 responses.	+ Myelin levels + Regenerative properties − Inflammation	[59]
Placental MSC-derived EVs	Duchenne muscular dystrophy	In vitro and vivo	Decrease in IL.6, TNF-α, Collagen I and II, and TGF-β. Increased myogenin and utrophin.	+ Muscle differentiation + Utrophin expression − Inflammation − Fibrosis	[60]
Placental MSC-derived EVs	Graft-versus-host disease	In vitro	Decrease in CD14+ and CD16+ monocytes.	+ Corticosteroid therapy v Inflammation	[61]
Placental MSC-derived EVs	Myocardial infarction	In vivo	Suppression of pro-inflammatory IL-1β, IL-6, TNF-ɑ, and MCP-1, and macrophage polarization (M2 subsets).	+ Cardiac function + Myocardial infarct area − Inflammation − Gut dysbiosis	[62]

**Table 2:** Therapeutic advantages of EVs released by chorion MSCs.

EVs’ Source	Disease Model	In Vitro and/or In Vivo Experiments	Molecular Mechanism Proposed	Pathophysiological Effects	References
Chorionic plate MSC-EVs	Hepatic failure	In vitro and in vivo	Increase in C-reactive protein (CRP) and vascular endothelial growth factor (VEGF) expression and promotion of the Wnt signaling pathway.	+ Regeneration + Proliferation + Angiogenesis + Autophagy − Fibrosis	[78]
Chorionic plate MSC-EVs	Hepatic fibrosis	In vivo	Inhibition of the expression of Smoothened (Smo) and downregulation of hedgehog (Hh) signaling.	+ Regenerative activity − Fibrosis	[79]
Chorionic plate MSC-EVs	Auricle ischemic injury	In vitro and in vivo	Increase in Tie2 and Ang2.	+ Angiogenesis + Endothelial tube formation	[80]

**Table 3:** Therapeutic advantages of EVs released by amnio-chorionic membrane MSCs.

EVs’ Source	Diseases Model	In Vitro and/or In Vivo Experiments	Molecular Mechanism Proposed	Pathophysiological Effects	References
hAEC-derived EVs	Premature ovarian failure	In vitro and in vivo	Enhancing the PPAR and AMPK signaling pathways and activation of other biologic pathways.	+ Ovarian function − Granulosa cell apoptosis − Acute vascular injury − Primordial follicle activation	[89]
hAEC-derived EVs	Acute kidney injury	In vitro and in vivo	Upregulation of various signaling pathways, e.g., extracellular matrix organization, cell adhesion, and leukocyte migration.	+ Proliferation + Angiogenesis − Peritubular capillary loss − Apoptosis − Ischemia–reperfusion	[90]
hAEC-derived EVs	Diabetic wounds	In vitro and in vivo	Activation of the PI3K-AKT-mTOR pathway.	+ Proliferation and migration of HFBs + Revascularization + Collagen deposition - Inflammation	[91]
hAEC-derived EVs	Wound healing	In vivo	Stimulation of fibroblast proliferation and extracellular matrix (ECM) remodeling.	+ Fibroblast proliferation + Reorganization of collagen fibers + Acceleration of wound healing	[92]
hAEC-derived EVs	Chronic Liver Fibrosis	In vitro and in vivo	Downregulation of TGF-β1 signaling.	− Hepatic stellate cell number − Collagen production − Hepatic macrophage infiltration	[93]
hAEC-derived EVs	Oxidative stress in pregnancy	In vitro	Activation of ERK/MAPK, PI3K/AKT pathways, as well as the epithelial adherent junctions. Downregulation of the LPS/IL-1 pathway.	− Inflammation in the uterine cavity + Senescent fetal membrane cells + Term parturition	[94]
hAMSC-derived EVs	Orthopedic disorders	In vitro and preclinical studies	Activation of various pathways involved in inflammation and oxidative stress processes.	+ Tissue repair + Regenerative features − Inflammation	[95]
hAMSC-derived EVs	Hepatic fibrosis	In vitro	Activation of TGF-β1 signaling and deregulation of Col 1 and alpha-smooth actin (αSMA).	+ Anti-fibrotic activity − Pro-fibrotic markers (such as I collagen) − Growth of HSCs − Deposition of extracellular matrix	[96]
hCMSC-derived EVs	Osteoarthritic process	In vitro	Modulation of the activity, function, and composition of target cells.	+ Cartilage repair + Subchondral bone remodeling − Synovitis	[97]

**Table 4:** Therapeutic advantages of EVs released by hAF MSCs.

EVs’ Source	Diseases Model	In Vitro and/or In Vivo Experiments	Molecular Mechanism Proposed	Pathophysiological Effects	References
hAFMSC-derived EVs	Skeletal muscle atrophy	In vivo	Modulation of the expression of inflammatory (Il-1α, Il-6, and Il-4) and pro-resolving (Il-10) cytokines.	+ Muscle strength + Supporting long-term regeneration − Inflammation − Muscle degeneration	[109]
hAFMSC-derived EVs	Ischemic stroke	In vitro	Activation of the neurotrophic pathways BDNF/TrkB and the survival pathways PI3K/Akt and ERK5, as well as by the suppression of the death pathways (p75/JNK).	+ Cell survival + Neuronal plasticity + Neural replacement − Cognitive loss − Apoptosis − Inflammation − Oxidative stress	[110]
hAFMSC-derived EVs	Osteoarthritis	In vitro and in vivo	Increase in anti-inflammatory cytokines (e.g., IL-10) and decrease in pro-inflammatory cytokines (e.g., IL-1b, IL-6, TNF-α, and IL-12).	+ Cartilage repair + Cell proliferation + Cell migration + Cartilage matrix synthesis − Degeneration of cartilage	[111]
hAFMSC-derived EVs	Ischemic myocardial	In vitro and in vivo	Activation of intracellular Ca2+ signals and activation of downstream Ca2+-dependent processes.	+ Cardioprotection + Pro-angiogenic action + Proliferation − Apoptosis	[112,113]
hAFMSC-derived EVs	Cutaneous injury	In vivo	Inhibition of TGF-βR1 and TGF-βR2 expression.	+ Wound healing rate + Regeneration levels of hair follicles, nerves, and vessels + Natural distribution of collagen fibers − Fibroblast differentiation − Fibrotic scarring	[114]
hAFMSC-derived EVs	Necrotizing enterocolitis	In vitro and vivo	Activation of Wnt/β-catenin pathway signaling.	+ Cellular regeneration + Mitigating damage in intestinal tissue − Acting against intestinal injury − Mucosal inflammation	[115]
hAFMSC-derived EVs	Premature ovarian failure	In vitro and vivo	Negative modulation of cell apoptosis and positive regulation of cell survival.	+ Survival of granulosa cells + Apoptotic resistance	[116]

**Table 5:** Therapeutic advantages of EVs released by umbilical cord MSCs.

EVs’ Source	Diseases Model	In Vitro and/or In Vivo Experiments	Molecular Mechanism Proposed	Pathophysiological Effects	References
hUCMSC-derived EVs	Liver fibrosis	In vivo	Inactivation of TGF-β1/Smad signaling pathway.	+ Tissue repair + Liver function − Reduction in collagen deposition − Inflammation	[141]
hUCMSC-derived EVs	Acute kidney injury	In vitro and in vivo	Activation of ERK1/2 pathway signaling and inhibition of p38 MAPK pathway signaling.	+ Kidney tubular cell proliferation − Necrosis of the proximal epithelium − Apoptosis	[142]
hWJMSC-derived EVs	Acute kidney injury	In vitro and in vivo	Activation of Nrf2/ARE pathway signaling.	+ Tissue repair − Apoptosis − Oxidative stress	[143]
hUCBMSC-derived EVs	Liver fibrosis	In vivo	Downregulation of the TGF-β-ID1 signaling pathway and regulation of the MMP/TIMP balance.	+ Liver function − Collagen production − HSC proliferation	[144]
hUCMSC-derived EVs	Inflammatory bowel disease	In vitro and in vivo	Regulating the expression of cytokines (decrease in TNF-α, IL-1β, and IL-6 but an increase of IL-10 in colon tissues and the spleen). Inhibition of the expression of IL-7 in macrophages.	− Infiltration of macrophages − Inflammation − Tissue injury	[145]
hUCMSC-derived EVs	Uveoretinitis	In vivo	Activation of MYD88-dependent signaling via Toll-like receptor (TLR) 4 ligands in monocytes, induction of the M2-like macrophage phenotype, and stimulation of CD4+ T cells.	+ Protection of retinal structure + Retinal function − Leukocyte infiltration − Inflammation	[146]
hUCMSC-derived EVs	Myocardial infarction	In vitro	Increase in Bcl-2 in cardiomyocytes and ATP levels. Decrease in oxidative stress and activation of PI3K/Akt pathway signaling.	+ Myocardium regeneration + Cell proliferation in the border zone + Angiogenesis − Cardiac fibrosis − Cardiomyocyte apoptosis	[147]
hUCMSC-derived EVs	Myocardial infarction	In vitro	Inhibition of SOX6 and the JNK3/caspase-3 pathway and activation of AKT.	+ Cardiac repair + Cardiac regeneration + Enhanced myocardial viability − Oxidative stress	[148]
hUCMSC-derived EVs	Endometrial injury	In vitro	Activation of the PTEN/AKT signaling pathway, as well as upregulation of Bcl-2 and downregulation of cleaved caspase-3.	+ Cell survival of damaged cells + Proliferation of damaged cells + Regeneration of tissue − apoptosis	[149]
hUCMSC-derived EVs	Atopic dermatitis	In vitro and in vivo	Suppression of T cell activation via reductions in the levels of IFN-γ (Th1 cell marker), IL-17 (Th17 cell marker), IL-4, IL-5, and IL-13 (Th2 cell marker), and B-cell-mediated serum IgE. Inhibition of NF-κB activity.	− Atopic histopathological symptoms − Allergic responses systemically − Inflammation − Immune responses	[150]
hUCMSC-derived EVs	Wound healing	In vitro	Enrichment in VEGF-A, FGF-2, HGF, and PDGF-BB and TGF-β molecules.	+ Dermal fibroblast proliferation + Keratinocyte proliferation	[151]
hUCMSC-derived EVs	Traumatic spinal cord injury	In vitro and in vivo	Decrease in pro-inflammatory cytokines, such as IL-1β and IL-6.	+ Long-term regenerative processes − Inflammation − Scarring activity	[152]
hWJMSC-derived EVs	Hypoxic–ischemic insult	In vitro	Regulation of caspase 3 (Casp3) transcription.	+ Neuroprotection + Neuroregeneration − Apoptosis − Neurodegeneration	[153]
hUCMSC-derived EVs	Lung cancer	In vitro	Reduction in PTEN protein expression by transferring miR-410.	+ Migration + Growth + Metastasis	[154]
hUCMSC-Ederived EVs	Pancreatic ductal adenocarcinoma	In vitro and in vivo	Downregulation of Smad3 and the mesenchymal marker N-cadherin. Upregulation of Bax.	+ Apoptosis + Cell cycle arrest − Cell proliferation − Invasion	[155]
hUCMSC-Ederived EVs	Breast cancer	In vitro and in vivo	Downregulation of protein levels of E-cadherin and Bax, as well as downregulation of protein levels of N-cadherin, vimentin, Bcl2, and Bcl-xl.	+ Apoptosis − Cell proliferation − Invasion − Migration	[156]
hWJMSC-derived EVs	Bladder carcinoma	In vitro and in vivo	Downregulation of the phosphorylation of Akt protein kinase and activation of p53/p21 and caspase 3.	+ Apoptosis − Cell proliferation − Metastasis	[157]
UCBMSC-derived EVs	Autoimmune encephalomyelitis	In vitro and in vivo	Inhibition of IL-2 signaling.	+ Suppression of T cell proliferation − Inflammation	[158]

## Abbreviations

AD: Alzheimer’s disease; AKI: acute kidney injury; AKT: protein chinasi B; alpha-SMA: alpha-smooth muscle actin; Ang2: angiopoietin 2; AβO: amyloid beta oligomers; BDNF: brain-derived neurotrophic factor; BM-MSCs: bone marrow mesenchymal stromal cells; CNS: central nervous system: CRP: C-reactive protein; CTB: cytophoblast; cGVHD: chronic graft-versus-host disease; dMSC: decidua mesenchymal stromal cells; ECM: extracellular matrix; eEVT: external extravillous trophoblast; ESCs: embryonic stem cells; EVs: extracellular vesicles; EVT: extravillous trophoblast; FGF: fibroblast growth factors; GFAP: glial fibrillary acidic protein; hAEC: human amniotic epithelial cells; hAF-MSCs: human amniotic fluid mesenchymal stromal cells; hAC-MSCs: human amnio-chorion mesenchymal stromal cells; hCTC: human chorionic trophoblast; hD-MSCs: human decidua mesenchymal stromal cells; hCP: human chorionic plate; hCTB-MSCs: human cytotrophoblast mesenchymal stromal cells; hCV-MSCs: human chorionic villi mesenchymal stromal cells; hCL-MSCs: human chorion laeve mesenchymal stromal cells; HFBs: human fibroblast; HGF: hepatocyte growth factor; Hh: hedgehog; hLF: human lung fibroblast; hPAM: human placental amniotic membrane; hRAM: human reflex amniotic membrane; hSTB-MSCs: human syncytiotrophoblast mesenchymal stromal cells; hUC-MSCs: human umbilical cord mesenchymal stromal cells; HUVEC: human umbilical vein endothelial cells; iEVT: inner extravillous trophoblast: IL: interleukin; IFN-α: interferon alpha; LPS: lipopolysaccharide binding protein; MCP1: monocyte chemoattractant protein-1; MSCs: mesenchymal stromal cells; mTOR: mammalian target of rapamycin; MyD88: myeloid differentiation primary response 88; NEC: necrotizing enterocolitis; PALP: alkaline phosphatase; PDGF: platelet-derived growth factor; TLR: toll-like receptors; PI3K: fosfatidilinositol-3-chinasi; POF: premature ovarian failure; PPAR: peroxisome proliferator-activated receptors; NRF2: nuclear factor erythroid 2–related factor 2; keap1: ECH associated protein 1; PAX6: paired Box 6; PnD: perinatal derivates; SCI: spinal cord injury; Smo: smoothened; SSEA4: expression of stage-specific embryonic antigen-4; STB: syncytiotrophoblast; Tie: angiopoietin-1 receptor; TGF-β1: transforming growth factor beta 1; TNF-α: tumor necrosis factor; TRA-1-60: T cell receptor alpha locus; VEGF: vascular endothelial growth factor; Wnt: wingless-related integration site; WJ-MSCs: Wharton’s jelly mesenchymal stromal cells.

## References

[1] Li S., Wang J., Jiang B., Jiang J., Luo L., Zheng B., Si W. (2022). Mesenchymal stem cells derived from different perinatal tissues donated by same donors manifest variant performance on the acute liver failure model in mouse. Stem Cell Res. Ther..

[2] Beeravolu N., McKee C., Alamri A., Mikhael S., Brown C., Perez-Cruet M., Chaudhry G.R. (2017). Isolation and Characterization of Mesenchymal Stromal Cells from Human Umbilical Cord and Fetal Placenta. J. Vis. Exp..

[3] Wu M., Zhang R., Zou Q., Chen Y., Zhou M., Li X., Ran R., Chen Q. (2018). Comparison of the Biological Characteristics of Mesenchymal Stem Cells Derived from the Human Placenta and Umbilical Cord. Sci. Rep..

[4] Caplan A.I., Dennis J.E. (2006). Mesenchymal stem cells as trophic mediators. J. Cell Biochem..

[5] Silini A.R., Di Pietro R., Lang-Olip I., Alviano F., Banerjee A., Basile M., Borutinskaite V., Eissner G., Gellhaus A., Giebel B. (2020). Where Do We Stand? A Roadmap of the Human Placenta and Consensus for Tissue and Cell Nomenclature. Front. Bioeng. Biotechnol..

[6] Alberti G., Russo E., Corrao S., Anzalone R., Kruzliak P., Miceli V., Conaldi P.G., Di Gaudio F., La Rocca G. (2022). Current Perspectives on Adult Mesenchymal Stromal Cell-Derived Extracellular Vesicles: Biological Features and Clinical Indications. Biomedicines.

[7] Russo E., Corrao S., Di Gaudio F., Alberti G., Caprnda M., Kubatka P., Kruzliak P., Miceli V., Conaldi P.G., Borlongan C.V. (2023). Facing the Challenges in the COVID-19 Pandemic Era: From Standard Treatments to the Umbilical Cord-Derived Mesenchymal Stromal Cells as a New Therapeutic Strategy. Cells.

[8] Lo Nigro A., Gallo A., Bulati M., Vitale G., Paini D.S., Pampalone M., Galvagno D., Conaldi P.G., Miceli V. (2021). Amnion-Derived Mesenchymal Stromal/Stem Cell Paracrine Signals Potentiate Human Liver Organoid Differentiation: Translational Implications for Liver Regeneration. Front. Med..

[9] Miceli V., Bertani A. (2022). Mesenchymal Stromal/Stem Cells and Their Products as a Therapeutic Tool to Advance Lung Transplantation. Cells.

[10] Miceli V., Bertani A., Chinnici C.M., Bulati M., Pampalone M., Amico G., Carcione C., Schmelzer E., Gerlach J.C., Conaldi P.G. (2021). Conditioned Medium from Human Amnion-Derived Mesenchymal Stromal/Stem Cells Attenuating the Effects of Cold Ischemia-Reperfusion Injury in an In Vitro Model Using Human Alveolar Epithelial Cells. Int. J. Mol. Sci..

[11] Miceli V., Bulati M., Iannolo G., Zito G., Gallo A., Conaldi P.G. (2021). Therapeutic Properties of Mesenchymal Stromal/Stem Cells: The Need of Cell Priming for Cell-Free Therapies in Regenerative Medicine. Int. J. Mol. Sci..

[12] Doyle L.M., Wang M.Z. (2019). Overview of Extracellular Vesicles, Their Origin, Composition, Purpose, and Methods for Exosome Isolation and Analysis. Cells.

[13] Heusermann W., Hean J., Trojer D., Steib E., von Bueren S., Graff-Meyer A., Genoud C., Martin K., Pizzato N., Voshol J. (2016). Exosomes surf on filopodia to enter cells at endocytic hot spots, traffic within endosomes, and are targeted to the ER. J. Cell Biol..

[14] Zaborowski M.P., Balaj L., Breakefield X.O., Lai C.P. (2015). Extracellular Vesicles: Composition, Biological Relevance, and Methods of Study. Bioscience.

[15] Cargnoni A., Papait A., Masserdotti A., Pasotti A., Stefani F.R., Silini A.R., Parolini O. (2021). Extracellular Vesicles From Perinatal Cells for Anti-inflammatory Therapy. Front. Bioeng. Biotechnol..

[16] Alberti G., Sánchez-López C.M., Andres A., Santonocito R., Campanella C., Cappello F., Marcilla A. (2021). Molecular Profile Study of Extracellular Vesicles for the Identification of Useful Small “Hit” in Cancer Diagnosis. Appl. Sci..

[17] Tkach M., Théry C. (2016). Communication by extracellular vesicles: Where we are and where we need to go. Cell.

[18] Alberti G., Mazzola M., Gagliardo C., Pitruzzella A., Fucarini A., Giammanco M., Tomasello G., Carini F. (2021). Extracellular vesicles derived from gut microbiota in inflammatory bowel disease and colorectal cancer. Biomed. Pap. Med. Fac. Univ. Palacky. Olomouc Czech Repub..

[19] Théry C., Witwer K.W., Aikawa E., Alcaraz M.J., Anderson J.D., Andriantsitohaina R., Antoniou A., Arab T., Archer F., Atkin-Smith G.K. (2018). Minimal information for studies of extracellular vesicles 2018 (MISEV2018): A position statement of the International Society for Extracellular Vesicles and update of the MISEV2014 guidelines. J. Extracell. Vesicles.

[20] Burton G.J., Jauniaux E. (2018). Development of the Human Placenta and Fetal Heart: Synergic or Independent?. Front. Physiol..

[21] Cross J.C., Werb Z., Fisher S.J. (1994). Implantation and the placenta: Key pieces of the development puzzle. Science.

[22] Plitman Mayo R. (2018). Advances in Human Placental Biomechanics. Comput. Struct. Biotechnol. J..

[23] Anzalone R., Lo Iacono M., Corrao S., Magno F., Loria T., Cappello F., Zummo G., Farina F., La Rocca G. (2010). New emerging potentials for human Wharton’s jelly mesenchymal stem cells: Immunological features and hepatocyte-like differentiative capacity. Stem Cells Dev..

[24] Portmann-Lanz C.B., Schoeberlein A., Huber A., Sager R., Malek A., Holzgreve W., Surbek D.V. (2006). Placental mesenchymal stem cells as potential autologous graft for pre- and perinatal neuroregeneration. Am. J. Obstet. Gynecol..

[25] La Rocca G., Anzalone R., Corrao S., Magno F., Loria T., Lo Iacono M., Di Stefano A., Giannuzzi P., Marasà L., Cappello F. (2009). Isolation and characterization of Oct-4+/HLA-G+ mesenchymal stem cells from human umbilical cord matrix: Differentiation potential and detection of new markers. Histochem. Cell Biol..

[26] Harrell C.R., Jovicic N., Djonov V., Arsenijevic N., Volarevic V. (2019). Mesenchymal Stem Cell-Derived Exosomes and Other Extracellular Vesicles as New Remedies in the Therapy of Inflammatory Diseases. Cells.

[27] Knöfler M., Haider S., Saleh L., Pollheimer J., Gamage T.K.J.B., James J. (2019). Human placenta and trophoblast development: Key molecular mechanisms and model systems. Cell. Mol. Life Sci..

[28] Vizoso F.J., Eiro N., Cid S., Schneider J., Perez-Fernandez R. (2017). Mesenchymal Stem Cell Secretome: Toward Cell-Free Therapeutic Strategies in Regenerative Medicine. Int. J. Mol. Sci..

[29] Thomi G., Surbek D., Haesler V., Joerger-Messerli M., Schoeberlein A. (2019). Exosomes derived from umbilical cord mesenchymal stem cells reduce microglia-mediated neuroinflammation in perinatal brain injury. Stem Cell Res. Ther..

[30] Gao F., Chiu S.M., Motan D.A., Zhang Z., Chen L., Ji H.L., Tse H.F., Fu Q.L., Lian Q. (2016). Mesenchymal stem cells and immunomodulation: Current status and future prospects. Cell Death Dis..

[31] Miceli V., Pampalone M., Vella S., Carreca A.P., Amico G., Conaldi P.G. (2019). Comparison of Immunosuppressive and Angiogenic Properties of Human Amnion-Derived Mesenchymal Stem Cells between 2D and 3D Culture Systems. Stem Cells Int..

[32] Wang J., Bonacquisti E.E., Brown A.D., Nguyen J. (2020). Boosting the Biogenesis and Secretion of Mesenchymal Stem Cell-Derived Exosomes. Cells.

[33] Revenfeld A.L., Bæk R., Nielsen M.H., Stensballe A., Varming K., Jørgensen M. (2014). Diagnostic and prognostic potential of extracellular vesicles in peripheral blood. Clin. Ther..

[34] Riazifar M., Pone E.J., Lötvall J., Zhao W. (2017). Stem Cell Extracellular Vesicles: Extended Messages of Regeneration. Annu. Rev. Pharmacol. Toxicol..

[35] Lai R.C., Arslan F., Lee M.M., Sze N.S., Choo A., Chen T.S., Salto-Tellez M., Timmers L., Lee C.N., El Oakley R.M. (2010). Exosome secreted by MSC reduces myocardial ischemia/reperfusion injury. Stem Cell Res..

[36] Antounians L., Tzanetakis A., Pellerito O., Catania V.D., Sulistyo A., Montalva L., McVey M.J., Zani A. (2019). The Regenerative Potential of Amniotic Fluid Stem Cell Extracellular Vesicles: Lessons Learned by Comparing Different Isolation Techniques. Sci. Rep..

[37] Wang Z.G., He Z.Y., Liang S., Yang Q., Cheng P., Chen A.M. (2020). Comprehensive proteomic analysis of exosomes derived from human bone marrow, adipose tissue, and umbilical cord mesenchymal stem cells. Stem Cell Res. Ther..

[38] Fang S., Xu C., Zhang Y., Xue C., Yang C., Bi H., Qian X., Wu M., Ji K., Zhao Y. (2016). Umbilical Cord-Derived Mesenchymal Stem Cell-Derived Exosomal MicroRNAs Suppress Myofibroblast Differentiation by Inhibiting the Transforming Growth Factor-β/SMAD2 Pathway During Wound Healing. Stem Cells Transl. Med..

[39] Zou X., Zhang G., Cheng Z., Yin D., Du T., Ju G., Miao S., Liu G., Lu M., Zhu Y. (2014). Microvesicles derived from human Wharton’s Jelly mesenchymal stromal cells ameliorate renal ischemia-reperfusion injury in rats by suppressing CX3CL1. Stem Cell Res. Ther..

[40] Lange-Consiglio A., Lazzari B., Perrini C., Pizzi F., Stella A., Cremonesi F., Capra E. (2018). MicroRNAs of Equine Amniotic Mesenchymal Cell-derived Microvesicles and Their Involvement in Anti-inflammatory Processes. Cell Transpl..

[41] Lange-Consiglio A., Lazzari B., Pizzi F., Stella A., Girani A., Quintè A., Cremonesi F., Capra E. (2018). Different Culture Times Affect MicroRNA Cargo in Equine Amniotic Mesenchymal Cells and Their Microvesicles. Tissue Eng. C.

[42] Meng X., Xue M., Xu P., Hu F., Sun B., Xiao Z. (2017). MicroRNA profiling analysis revealed different cellular senescence mechanisms in human mesenchymal stem cells derived from different origin. Genomics.

[43] Bulati M., Miceli V., Gallo A., Amico G., Carcione C., Pampalone M., Conaldi P.G. (2020). The Immunomodulatory Properties of the Human Amnion-Derived Mesenchymal Stromal/Stem Cells Are Induced by INF-γ Produced by Activated Lymphomonocytes and Are Mediated by Cell-To-Cell Contact and Soluble Factors. Front. Immunol..

[44] Zou X.Y., Yu Y., Lin S., Zhong L., Sun J., Zhang G., Zhu Y. (2018). Comprehensive miRNA Analysis of Human Umbilical Cord-Derived Mesenchymal Stromal Cells and Extracellular Vesicles. Kidney Blood Press. Res..

[45] Fujita Y., Kadota T., Araya J., Ochiya T., Kuwano K. (2018). Clinical Application of Mesenchymal Stem Cell-Derived Extracellular Vesicle-Based Therapeutics for Inflammatory Lung Diseases. J. Clin. Med..

[46] Zhang S., Mesalam A., Joo M.D., Lee K.L., Hwang J.Y., Xu L., Song S.H., Koh P.O., Yuan Y.G., Lv W. (2020). Matrix metalloproteinases improves trophoblast invasion and pregnancy potential in mice. Theriogenology.

[47] Abomaray F.M., Al Jumah M.A., Alsaad K.O., Jawdat D., Al Khaldi A., AlAskar A.S., Al Harthy S., Al Subayyil A.M., Khatlani T., Alawad A.O. (2016). Phenotypic and Functional Characterization of Mesenchymal Stem/Multipotent Stromal Cells from Decidua Basalis of Human Term Placenta. Stem Cells Int..

[48] Macias M.I., Grande J., Moreno A., Domínguez I., Bornstein R., Flores A.I. (2010). Isolation and characterization of true mesenchymal stem cells derived from human term decidua capable of multilineage differentiation into all 3 embryonic layers. Am. J. Obstet. Gynecol..

[49] Rice G.E., Scholz-Romero K., Sweeney E., Peiris H., Kobayashi M., Duncombe G., Mitchell M.D., Salomon C. (2015). The Effect of Glucose on the Release and Bioactivity of Exosomes from First Trimester Trophoblast Cells. J. Clin. Endocrinol. Metab..

[50] Salomon C., Kobayashi M., Ashman K., Sobrevia L., Mitchell M.D., Rice G.E. (2013). Hypoxia-induced changes in the bioactivity of cytotrophoblast-derived exosomes. PLoS ONE.

[51] Sarker S., Scholz-Romero K., Perez A., Illanes S.E., Mitchell M.D., Rice G.E., Salomon C. (2014). Placenta-derived exosomes continuously increase in maternal circulation over the first trimester of pregnancy. J. Transl. Med..

[52] Salomon C., Torres M.J., Kobayashi M., Scholz-Romero K., Sobrevia L., Dobierzewska A., Illanes S.E., Mitchell M.D., Rice G.E. (2014). A gestational profile of placental exosomes in maternal plasma and their effects on endothelial cell migration. PLoS ONE.

[53] Salomon C., Guanzon D., Scholz-Romero K., Longo S., Correa P., Illanes S.E., Rice G.E. (2017). Placental Exosomes as Early Biomarker of Preeclampsia: Potential Role of Exosomal MicroRNAs Across Gestation. J. Clin. Endocrinol. Metab..

[54] Herrera-Van Oostdam A.S., Toro-Ortíz J.C., López J.A., Noyola D.E., García-López D.A., Durán-Figueroa N.V., Martínez-Martínez E., Portales-Pérez D.P., Salgado-Bustamante M., López-Hernández Y. (2020). Placental exosomes isolated from urine of patients with gestational diabetes exhibit a differential profile expression of microRNAs across gestation. Int. J. Mol. Med..

[55] Cao H., Cheng Y., Gao H., Zhuang J., Zhang W., Bian Q., Wang F., Du Y., Li Z., Kong D. (2020). In Vivo Tracking of Mesenchymal Stem Cell-Derived Extracellular Vesicles Improving Mitochondrial Function in Renal Ischemia-Reperfusion Injury. ACS Nano.

[56] Zhang K., Chen S., Sun H., Wang L., Li H., Zhao J., Zhang C., Li N., Guo Z., Han Z. (2020). In vivo two-photon microscopy reveals the contribution of Sox9^+^ cell to kidney regeneration in a mouse model with extracellular vesicle treatment. J. Biol. Chem..

[57] Duan L., Huang H., Zhao X., Zhou M., Chen S., Wang C., Han Z., Han Z.C., Guo Z., Li Z. (2020). Extracellular vesicles derived from human placental mesenchymal stem cells alleviate experimental colitis in mice by inhibiting inflammation and oxidative stress. Int. J. Mol. Med..

[58] Zhou W., Silva M., Feng C., Zhao S., Liu L., Li S., Zhong J., Zheng W. (2021). Exosomes derived from human placental mesenchymal stem cells enhanced the recovery of spinal cord injury by activating endogenous neurogenesis. Stem Cell Res. Ther..

[59] Clark K., Zhang S., Barthe S., Kumar P., Pivetti C., Kreutzberg N., Reed C., Wang Y., Paxton Z., Farmer D. (2019). Placental Mesenchymal Stem Cell-Derived Extracellular Vesicles Promote Myelin Regeneration in an Animal Model of Multiple Sclerosis. Cells.

[60] Bier A., Berenstein P., Kronfeld N., Morgoulis D., Ziv-Av A., Goldstein H., Kazimirsky G., Cazacu S., Meir R., Popovtzer R. (2018). Placenta-derived mesenchymal stromal cells and their exosomes exert therapeutic effects in Duchenne muscular dystrophy. Biomaterials.

[61] Norooznezhad A.H., Yarani R., Payandeh M., Hoseinkhani Z., Kiani S., Taghizadeh E., Thakor A.S., Mansouri K. (2022). Human placental mesenchymal stromal cell-derived exosome-enriched extracellular vesicles for chronic cutaneous graft-versus-host disease: A case report. J. Cell. Mol. Med..

[62] Yang L., Wang T., Zhang X., Zhang H., Yan N., Zhang G., Yan R., Li Y., Yu J., He J. (2022). Exosomes derived from human placental mesenchymal stem cells ameliorate myocardial infarction via anti-inflammation and restoring gut dysbiosis. BMC Cardiovasc. Disord..

[63] Ramachandran V., Kolli S.S., Strowd L.C. (2019). Review of Graft-Versus-Host Disease. Dermatol. Clin..

[64] Maître J.L. (2017). Mechanics of blastocyst morphogenesis. Biol. Cell.

[65] Baczyk D., Drewlo S., Proctor L., Dunk C., Lye S., Kingdom J. (2009). Glial cell missing-1 transcription factor is required for the differentiation of the human trophoblast. Cell Death Differ..

[66] Moffett A., Loke C. (2006). Immunology of placentation in eutherian mammals. Nat. Rev. Immunol..

[67] Gauster M., Moser G., Wernitznig S., Kupper N., Huppertz B. (2022). Early human trophoblast development: From morphology to function. Cell. Mol. Life Sci..

[68] Sabapatha A., Gercel-Taylor C., Taylor D.D. (2006). Specific isolation of placenta-derived exosomes from the circulation of pregnant women and their immunoregulatory consequences. Am. J. Reprod. Immunol..

[69] Tannetta D., Mackeen M., Kessler B., Sargent I., Redman C. (2012). OS045. Multi-dimensional protein identification technology analysis of syncytiotrophoblast vesicles released from perfused preeclampsia placentas. Pregnancy Hypertens..

[70] Tannetta D.S., Dragovic R.A., Gardiner C., Redman C.W., Sargent I.L. (2013). Characterisation of syncytiotrophoblast vesicles in normal pregnancy and pre-eclampsia: Expression of Flt-1 and endoglin. PLoS ONE.

[71] Taylor S.K., Houshdaran S., Robinson J.F., Gormley M.J., Kwan E.Y., Kapidzic M., Schilling B., Giudice L.C., Fisher S.J. (2020). Cytotrophoblast extracellular vesicles enhance decidual cell secretion of immune modulators via TNFα. Development.

[72] Parolini O., Alviano F., Bagnara G.P., Bilic G., Bühring H.J., Evangelista M., Hennerbichler S., Liu B., Magatti M., Mao N. (2008). Concise review: Isolation and characterization of cells from human term placenta: Outcome of the first international Workshop on Placenta Derived Stem Cells. Stem Cells.

[73] Dominici M., Le Blanc K., Mueller I., Slaper-Cortenbach I., Marini F., Krause D., Deans R., Keating A., Prockop D., Horwitz E. (2006). Minimal criteria for defining multipotent mesenchymal stromal cells. The International Society for Cellular Therapy position statement. Cytotherapy.

[74] Yen B.L., Huang H.I., Chien C.C., Jui H.Y., Ko B.S., Yao M., Shun C.T., Yen M.L., Lee M.C., Chen Y.C. (2005). Isolation of multipotent cells from human term placenta. Stem Cells.

[75] Huang Q., Yang Y., Luo C., Wen Y., Liu R., Li S., Chen T., Sun H., Tang L. (2019). An efficient protocol to generate placental chorionic plate-derived mesenchymal stem cells with superior proliferative and immunomodulatory properties. Stem Cell Res. Ther..

[76] Ma J., Wu J., Han L., Jiang X., Yan L., Hao J., Wang H. (2019). Comparative analysis of mesenchymal stem cells derived from amniotic membrane, umbilical cord, and chorionic plate under serum-free condition. Stem Cell Res. Ther..

[77] Torre P., Flores A.I. (2020). Current Status and Future Prospects of Perinatal Stem Cells. Genes.

[78] Jun J.H., Kim J.Y., Choi J.H., Lim J.Y., Kim K., Kim G.J. (2020). Exosomes from Placenta-Derived Mesenchymal Stem Cells Are Involved in Liver Regeneration in Hepatic Failure Induced by Bile Duct Ligation. Stem Cells Int..

[79] Hyun J., Wang S., Kim J., Kim G.J., Jung Y. (2015). MicroRNA125b-mediated Hedgehog signaling influences liver regeneration by chorionic plate-derived mesenchymal stem cells. Sci. Rep..

[80] Komaki M., Numata Y., Morioka C., Honda I., Tooi M., Yokoyama N., Ayame H., Iwasaki K., Taki A., Oshima N. (2017). Exosomes of human placenta-derived mesenchymal stem cells stimulate angiogenesis. Stem Cell Res. Ther..

[81] Truong G., Guanzon D., Kinhal V., Elfeky O., Lai A., Longo S., Nuzhat Z., Palma C., Scholz-Romero K., Menon R. (2017). Oxygen tension regulates the miRNA profile and bioactivity of exosomes released from extravillous trophoblast cells-Liquid biopsies for monitoring complications of pregnancy. PLoS ONE.

[82] Centurione L., Passaretta F., Centurione M.A., Munari S., Vertua E., Silini A., Liberati M., Parolini O., Di Pietro R. (2018). Mapping of the Human Placenta: Experimental Evidence of Amniotic Epithelial Cell Heterogeneity. Cell Transpl..

[83] Bilic G., Zeisberger S.M., Mallik A.S., Zimmermann R., Zisch A.H. (2008). Comparative characterization of cultured human term amnion epithelial and mesenchymal stromal cells for application in cell therapy. Cell Transpl..

[84] Alviano F., Fossati V., Marchionni C., Arpinati M., Bonsi L., Franchina M., Lanzoni G., Cantoni S., Cavallini C., Bianchi F. (2007). Term Amniotic membrane is a high throughput source for multipotent Mesenchymal Stem Cells with the ability to differentiate into endothelial cells in vitro. BMC Dev. Biol..

[85] Pampalone M., Corrao S., Amico G., Vitale G., Alduino R., Conaldi P.G., Pietrosi G. (2021). Human Amnion-Derived Mesenchymal Stromal Cells in Cirrhotic Patients with Refractory Ascites: A Possible Anti-Inflammatory Therapy for Preventing Spontaneous Bacterial Peritonitis. Stem Cell Rev. Rep..

[86] Soncini M., Vertua E., Gibelli L., Zorzi F., Denegri M., Albertini A., Wengler G.S., Parolini O. (2007). Isolation and characterization of mesenchymal cells from human fetal membranes. J. Tissue Eng. Regen. Med..

[87] Weidinger A., Poženel L., Wolbank S., Banerjee A. (2021). Sub-Regional Differences of the Human Amniotic Membrane and Their Potential Impact on Tissue Regeneration Application. Front. Bioeng. Biotechnol..

[88] Hadley E.E., Sheller-Miller S., Saade G., Salomon C., Mesiano S., Taylor R.N., Taylor B.D., Menon R. (2018). Amnion epithelial cell-derived exosomes induce inflammatory changes in uterine cells. Am. J. Obstet. Gynecol..

[89] Zhang Q., Sun J., Huang Y., Bu S., Guo Y., Gu T., Li B., Wang C., Lai D. (2019). Human Amniotic Epithelial Cell-Derived Exosomes Restore Ovarian Function by Transferring MicroRNAs against Apoptosis. Mol. Therapy Nucleic Acids.

[90] Bai K., Li X., Zhong J., Ng E.H.Y., Yeung W.S.B., Lee C.L., Chiu P.C.N. (2021). Placenta-Derived Exosomes as a Modulator in Maternal Immune Tolerance During Pregnancy. Front. Immunol..

[91] Wei P., Zhong C., Yang X., Shu F., Xiao S., Gong T., Luo P., Li L., Chen Z., Zheng Y. (2020). Exosomes derived from human amniotic epithelial cells accelerate diabetic wound healing via PI3K-AKT-mTOR-mediated promotion in angiogenesis and fibroblast function. Burn. Trauma.

[92] Zhao B., Li X., Shi X., Shi X., Zhang W., Wu G., Wang X., Su L., Hu D. (2018). Exosomal MicroRNAs Derived from Human Amniotic Epithelial Cells Accelerate Wound Healing by Promoting the Proliferation and Migration of Fibroblasts. Stem Cells Int..

[93] Alhomrani M., Correia J., Zavou M., Leaw B., Kuk N., Xu R., Saad M.I., Hodge A., Greening D.W., Lim R. (2017). The Human Amnion Epithelial Cell Secretome Decreases Hepatic Fibrosis in Mice with Chronic Liver Fibrosis. Front. Pharmacol..

[94] Sheller S., Papaconstantinou J., Urrabaz-Garza R., Richardson L., Saade G., Salomon C., Menon R. (2016). Amnion-Epithelial-Cell-Derived Exosomes Demonstrate Physiologic State of Cell under Oxidative Stress. PLoS ONE.

[95] Ragni E., Papait A., Perucca Orfei C., Silini A.R., Colombini A., Viganò M., Libonati F., Parolini O., de Girolamo L. (2021). Amniotic membrane-mesenchymal stromal cells secreted factors and extracellular vesicle-miRNAs: Anti-inflammatory and regenerative features for musculoskeletal tissues. Stem Cells Transl. Med..

[96] Mao Y., Jacob V., Singal A., Lei S., Park M.S., Lima M., Li C., Dhall S., Sathyamoorthy M., Kohn J. (2021). Exosomes Secreted from Amniotic Membrane Contribute to Its Anti-Fibrotic Activity. Int. J. Mol. Sci..

[97] Janockova J., Matejova J., Moravek M., Homolova L., Slovinska L., Nagyova A., Rak D., Sedlak M., Harvanova D., Spakova T. (2021). Small Extracellular Vesicles Derived from Human Chorionic MSCs as Modern Perspective towards Cell-Free Therapy. Int. J. Mol. Sci..

[98] Tan J.L., Lau S.N., Leaw B., Nguyen H., Salamonsen L.A., Saad M.I., Chan S.T., Zhu D., Krause M., Kim C. (2018). Amnion Epithelial Cell-Derived Exosomes Restrict Lung Injury and Enhance Endogenous Lung Repair. Stem Cells Transl. Med..

[99] Hoyes A.D. (1975). Structure and function of the amnion. Obstet. Gynecol. Annu..

[100] Modena A.B., Fieni S. (2004). Amniotic fluid dynamics. Acta Biomed..

[101] Bhatti G., Romero R., Gomez-Lopez N., Chaiworapongsa T., Jung E., Gotsch F., Pique-Regi R., Pacora P., Hsu C.D., Kavdia M. (2022). The amniotic fluid proteome changes with gestational age in normal pregnancy: A cross-sectional study. Sci. Rep..

[102] Casciaro F., Zia S., Forcato M., Zavatti M., Beretti F., Bertucci E., Zattoni A., Reschiglian P., Alviano F., Bonsi L. (2021). Unravelling Heterogeneity of Amplified Human Amniotic Fluid Stem Cells Sub-Populations. Cells.

[103] Tsai M.S., Lee J.L., Chang Y.J., Hwang S.M. (2004). Isolation of human multipotent mesenchymal stem cells from second-trimester amniotic fluid using a novel two-stage culture protocol. Hum. Reprod..

[104] Moraghebi R., Kirkeby A., Chaves P., Rönn R.E., Sitnicka E., Parmar M., Larsson M., Herbst A., Woods N.B. (2017). Term amniotic fluid: An unexploited reserve of mesenchymal stromal cells for reprogramming and potential cell therapy applications. Stem Cell Res. Ther..

[105] Spitzhorn L.S., Rahman M.S., Schwindt L., Ho H.T., Wruck W., Bohndorf M., Wehrmeyer S., Ncube A., Beyer I., Hagenbeck C. (2017). Isolation and Molecular Characterization of Amniotic Fluid-Derived Mesenchymal Stem Cells Obtained from Caesarean Sections. Stem Cells Int..

[106] Di Trapani M., Bassi G., Fontana E., Giacomello L., Pozzobon M., Guillot P.V., De Coppi P., Krampera M. (2015). Immune regulatory properties of CD117(pos) amniotic fluid stem cells vary according to gestational age. Stem Cells Dev..

[107] Schiavo A.A., Franzin C., Albiero M., Piccoli M., Spiro G., Bertin E., Urbani L., Visentin S., Cosmi E., Fadini G.P. (2015). Endothelial properties of third-trimester amniotic fluid stem cells cultured in hypoxia. Stem Cell Res. Ther..

[108] Dorazehi F., Nabiuni M., Jalali H. (2018). Potential Use of Amniotic Membrane-Derived Scaffold for Cerebrospinal Fluid Applications. Int. J. Mol. Cell. Med..

[109] Balbi C., Piccoli M., Barile L., Papait A., Armirotti A., Principi E., Reverberi D., Pascucci L., Becherini P., Varesio L. (2017). First Characterization of Human Amniotic Fluid Stem Cell Extracellular Vesicles as a Powerful Paracrine Tool Endowed with Regenerative Potential. Stem Cells Transl. Med..

[110] Castelli V., Antonucci I., d’Angelo M., Tessitore A., Zelli V., Benedetti E., Ferri C., Desideri G., Borlongan C., Stuppia L. (2021). Neuroprotective effects of human amniotic fluid stem cells-derived secretome in an ischemia/reperfusion model. Stem Cells Transl. Med..

[111] Zavatti M., Beretti F., Casciaro F., Bertucci E., Maraldi T. (2020). Comparison of the therapeutic effect of amniotic fluid stem cells and their exosomes on monoiodoacetate-induced animal model of osteoarthritis. BioFactors.

[112] Balbi C., Lodder K., Costa A., Moimas S., Moccia F., van Herwaarden T., Rosti V., Campagnoli F., Palmeri A., De Biasio P. (2019). Supporting data on in vitro cardioprotective and proliferative paracrine effects by the human amniotic fluid stem cell secretome. Data Brief.

[113] Costa A., Balbi C., Garbati P., Palamà M.E.F., Reverberi D., De Palma A., Rossi R., Paladini D., Coviello D., De Biasio P. (2022). Investigating the Paracrine Role of Perinatal Derivatives: Human Amniotic Fluid Stem Cell-Extracellular Vesicles Show Promising Transient Potential for Cardiomyocyte Renewal. Front. Bioeng. Biotechnol..

[114] Zhang Y., Yan J., Liu Y., Chen Z., Li X., Tang L., Li J., Duan M., Zhang G. (2021). Human Amniotic Fluid Stem Cell-Derived Exosomes as a Novel Cell-Free Therapy for Cutaneous Regeneration. Front. Cell Dev. Biol..

[115] Li B., Lee C., O’Connell J.S., Antounians L., Ganji N., Alganabi M., Cadete M., Nascimben F., Koike Y., Hock A. (2020). Activation of Wnt signaling by amniotic fluid stem cell-derived extracellular vesicles attenuates intestinal injury in experimental necrotizing enterocolitis. Cell Death Dis..

[116] Xiao G.Y., Cheng C.C., Chiang Y.S., Cheng W.T., Liu I.H., Wu S.C. (2016). Exosomal miR-10a derived from amniotic fluid stem cells preserves ovarian follicles after chemotherapy. Sci. Rep..

[117] Beretti F., Zavatti M., Casciaro F., Comitini G., Franchi F., Barbieri V., La Sala G.B., Maraldi T. (2018). Amniotic fluid stem cell exosomes: Therapeutic perspective. BioFactors.

[118] Dixon C.L., Sheller-Miller S., Saade G.R., Fortunato S.J., Lai A., Palma C., Guanzon D., Salomon C., Menon R. (2018). Amniotic Fluid Exosome Proteomic Profile Exhibits Unique Pathways of Term and Preterm Labor. Endocrinology.

[119] Gebara N., Scheel J., Skovronova R., Grange C., Marozio L., Gupta S., Giorgione V., Caicci F., Benedetto C., Khalil A. (2022). Single extracellular vesicle analysis in human amniotic fluid shows evidence of phenotype alterations in preeclampsia. J. Extracell. Vesicles.

[120] Wharton T.W., Freer S. (1996). Adenographia.

[121] Nanaev A.K., Kohnen G., Milovanov A.P., Domogatsky S.P., Kaufmann P. (1997). Stromal differentiation and architecture of the human umbilical cord. Placenta.

[122] Davies J.E., Walker J.T., Keating A. (2017). Concise Review: Wharton’s Jelly: The Rich, but Enigmatic, Source of Mesenchymal Stromal Cells. Stem Cells Transl. Med..

[123] Russo E., Lee J.Y., Nguyen H., Corrao S., Anzalone R., La Rocca G., Borlongan C.V. (2020). Energy Metabolism Analysis of Three Different Mesenchymal Stem Cell Populations of Umbilical Cord Under Normal and Pathologic Conditions. Stem Cell Rev. Rep..

[124] Cozene B.M., Russo E., Anzalone R., Rocca G., Borlongan C.V. (2021). Mitochondrial activity of human umbilical cord mesenchymal stem cells. Brain Circ..

[125] Yang S.E., Ha C.W., Jung M., Jin H.J., Lee M., Song H., Choi S., Oh W., Yang Y.S. (2004). Mesenchymal stem/progenitor cells developed in cultures from UC blood. Cytotherapy.

[126] Thompson M., Mei S., Wolfe D., Champagne J., Fergusson D., Stewart D.J., Sullivan K.J., Doxtator E., Lalu M., English S.W. (2020). Cell therapy with intravascular administration of mesenchymal stromal cells continues to appear safe: An updated systematic review and meta-analysis. EClinicalMedicine.

[127] Karahuseyinoglu S., Cinar O., Kilic E., Kara F., Akay G.G., Demiralp D.O., Tukun A., Uckan D., Can A. (2007). Biology of stem cells in human umbilical cord stroma: In situ and in vitro surveys. Stem Cells.

[128] Corrao S., La Rocca G., Lo Iacono M., Corsello T., Farina F., Anzalone R. (2013). Umbilical cord revisited: From Wharton’s jelly myofibroblasts to mesenchymal stem cells. Histol. Histopathol..

[129] Lo Iacono M., Russo E., Anzalone R., Baiamonte E., Alberti G., Gerbino A., Maggio A., La Rocca G., Acuto S. (2018). Wharton’s Jelly Mesenchymal Stromal Cells Support the Expansion of Cord Blood-derived CD34^+^ Cells Mimicking a Hematopoietic Niche in a Direct Cell-cell Contact Culture System. Cell Transpl..

[130] Lo Iacono M., Anzalone R., La Rocca G., Baiamonte E., Maggio A., Acuto S. (2017). Wharton’s Jelly Mesenchymal Stromal Cells as a Feeder Layer for the Ex Vivo Expansion of Hematopoietic Stem and Progenitor Cells: A Review. Stem Cell Rev. Rep..

[131] Wang H., Yan X., Jiang Y., Wang Z., Li Y., Shao Q. (2018). The human umbilical cord stem cells improve the viability of OA degenerated chondrocytes. Mol. Med. Rep..

[132] Moretti P., Hatlapatka T., Marten D., Lavrentieva A., Majore I., Hass R., Kasper C. (2010). Mesenchymal stromal cells derived from human umbilical cord tissues: Primitive cells with potential for clinical and tissue engineering applications. Adv. Biochem. Eng. Biotechnol..

[133] Batsali A.K., Kastrinaki M.C., Papadaki H.A., Pontikoglou C. (2013). Mesenchymal stem cells derived from Wharton’s Jelly of the umbilical cord: Biological properties and emerging clinical applications. Curr. Stem Cell Res. Ther..

[134] Stefańska K., Bryl R., Hutchings G., Shibli J.A., Dyszkiewicz-Konwińska M. (2020). Human umbilical cord stem cells–the discovery, history and possible application. Med. J. Cell Biol..

[135] Huang Y.C., Parolini O., La Rocca G., Deng L. (2012). Umbilical cord versus bone marrow-derived mesenchymal stromal cells. Stem Cells Dev..

[136] La Rocca G., Lo Iacono M., Corsello T., Corrao S., Farina F., Anzalone R. (2013). Human Wharton’s jelly mesenchymal stem cells maintain the expression of key immunomodulatory molecules when subjected to osteogenic, adipogenic and chondrogenic differentiation in vitro: New perspectives for cellular therapy. Curr. Stem Cell Res. Ther..

[137] Jadalannagari S., Aljitawi O.S. (2015). Ectodermal differentiation of Wharton’s jelly mesenchymal stem cells for tissue engineering and regenerative medicine applications. Tissue Eng. Part B Rev..

[138] Kern S., Eichler H., Stoeve J., Klüter H., Bieback K. (2006). Comparative analysis of mesenchymal stem cells from bone marrow, umbilical cord blood, or adipose tissue. Stem Cells.

[139] Ragni E., Banfi F., Barilani M., Cherubini A., Parazzi V., Larghi P., Dolo V., Bollati V., Lazzari L. (2017). Extracellular Vesicle-Shuttled mRNA in Mesenchymal Stem Cell Communication. Stem Cells.

[140] Zhang B., Shen L., Shi H., Pan Z., Wu L., Yan Y., Zhang X., Mao F., Qian H., Xu W. (2016). Exosomes from Human Umbilical Cord Mesenchymal Stem Cells: Identification, Purification, and Biological Characteristics. Stem Cells Int..

[141] Li T., Yan Y., Wang B., Qian H., Zhang X., Shen L., Wang M., Zhou Y., Zhu W., Li W. (2013). Exosomes derived from human umbilical cord mesenchymal stem cells alleviate liver fibrosis. Stem Cells Dev..

[142] Zhou Y., Xu H., Xu W., Wang B., Wu H., Tao Y., Zhang B., Wang M., Mao F., Yan Y. (2013). Exosomes released by human umbilical cord mesenchymal stem cells protect against cisplatin-induced renal oxidative stress and apoptosis in vivo and in vitro. Stem Cell Res. Ther..

[143] Zhang G., Zou X., Huang Y., Wang F., Miao S., Liu G., Chen M., Zhu Y. (2016). Mesenchymal Stromal Cell-Derived Extracellular Vesicles Protect Against Acute Kidney Injury Through Anti-Oxidation by Enhancing Nrf2/ARE Activation in Rats. Kidney Blood Press. Res..

[144] Huang Y.J., Cao J., Lee C.Y., Wu Y.M. (2021). Umbilical cord blood plasma-derived exosomes as a novel therapy to reverse liver fibrosis. Stem Cell Res. Ther..

[145] Mao F., Wu Y., Tang X., Kang J., Zhang B., Yan Y., Qian H., Zhang X., Xu W. (2017). Exosomes Derived from Human Umbilical Cord Mesenchymal Stem Cells Relieve Inflammatory Bowel Disease in Mice. BioMed Res. Int..

[146] Bai L., Shao H., Wang H., Zhang Z., Su C., Dong L., Yu B., Chen X., Li X., Zhang X. (2017). Effects of Mesenchymal Stem Cell-Derived Exosomes on Experimental Autoimmune Uveitis. Sci. Rep..

[147] Zhao Y., Sun X., Cao W., Ma J., Sun L., Qian H., Zhu W., Xu W. (2015). Exosomes Derived from Human Umbilical Cord Mesenchymal Stem Cells Relieve Acute Myocardial Ischemic Injury. Stem Cells Int..

[148] Huang L., Yang L., Ding Y., Jiang X., Xia Z., You Z. (2020). Human umbilical cord mesenchymal stem cells-derived exosomes transfers microRNA-19a to protect cardiomyocytes from acute myocardial infarction by targeting SOX6. Cell Cycle.

[149] Wang J., Hu R., Xing Q., Feng X., Jiang X., Xu Y., Wei Z. (2020). Exosomes Derived from Umbilical Cord Mesenchymal Stem Cells Alleviate Mifepristone-Induced Human Endometrial Stromal Cell Injury. Stem Cells Int..

[150] Song J.Y., Kang H.J., Ju H.M., Park A., Park H., Hong J.S., Kim C.J., Shim J.Y., Yu J., Choi J. (2019). Umbilical cord-derived mesenchymal stem cell extracts ameliorate atopic dermatitis in mice by reducing the T cell responses. Sci. Rep..

[151] Hoang D.H., Nguyen T.D., Nguyen H.P., Nguyen X.H., Do P., Dang V.D., Dam P., Bui H., Trinh M.Q., Vu D.M. (2020). Differential Wound Healing Capacity of Mesenchymal Stem Cell-Derived Exosomes Originated from Bone Marrow, Adipose Tissue and Umbilical Cord Under Serum- and Xeno-Free Condition. Front. Mol. Biosci..

[152] Romanelli P., Bieler L., Scharler C., Pachler K., Kreutzer C., Zaunmair P., Jakubecova D., Mrowetz H., Benedetti B., Rivera F.J. (2019). Extracellular Vesicles Can Deliver Anti-inflammatory and Anti-scarring Activities of Mesenchymal Stromal Cells After Spinal Cord Injury. Front. Neurol..

[153] Joerger-Messerli M.S., Oppliger B., Spinelli M., Thomi G., di Salvo I., Schneider P., Schoeberlein A. (2018). Extracellular Vesicles Derived from Wharton’s Jelly Mesenchymal Stem Cells Prevent and Resolve Programmed Cell Death Mediated by Perinatal Hypoxia-Ischemia in Neuronal Cells. Cell Transpl..

[154] Dong L., Pu Y., Zhang L., Qi Q., Xu L., Li W., Wei C., Wang X., Zhou S., Zhu J. (2018). Human umbilical cord mesenchymal stem cell-derived extracellular vesicles promote lung adenocarcinoma growth by transferring miR-410. Cell Death Dis..

[155] Ding Y., Cao F., Sun H., Wang Y., Liu S., Wu Y., Cui Q., Mei W., Li F. (2019). Exosomes derived from human umbilical cord mesenchymal stromal cells deliver exogenous miR-145-5p to inhibit pancreatic ductal adenocarcinoma progression. Cancer Lett..

[156] Yuan L., Liu Y., Qu Y., Liu L., Li H. (2019). Exosomes Derived from MicroRNA-148b-3p-Overexpressing Human Umbilical Cord Mesenchymal Stem Cells Restrain Breast Cancer Progression. Front. Oncol..

[157] Wu S., Ju G.Q., Du T., Zhu Y.J., Liu G.H. (2013). Microvesicles derived from human umbilical cord Wharton’s jelly mesenchymal stem cells attenuate bladder tumor cell growth in vitro and in vivo. PLoS ONE.

[158] Kim S., Maeng J.Y., Hyun S.J., Sohn H.J., Kim S.Y., Hong C.H., Kim T.G. (2020). Extracellular vesicles from human umbilical cord blood plasma modulate interleukin-2 signaling of T cells to ameliorate experimental autoimmune encephalomyelitis. Theranostics.

[159] Zhai X., Chen K., Yang H., Li B., Zhou T., Wang H., Zhou H., Chen S., Zhou X., Wei X. (2021). Extracellular vesicles derived from CD73 modified human umbilical cord mesenchymal stem cells ameliorate inflammation after spinal cord injury. J. Nanobiotechnol..

[160] Zhou X., Li T., Chen Y., Zhang N., Wang P., Liang Y., Long M., Liu H., Mao J., Liu Q. (2019). Mesenchymal stem cell-derived extracellular vesicles promote the in vitro proliferation and migration of breast cancer cells through the activation of the ERK pathway. Int. J. Oncol..

[161] Zhao X., Wu X., Qian M., Song Y., Wu D., Zhang W. (2018). Knockdown of TGF-β1 expression in human umbilical cord mesenchymal stem cells reverts their exosome-mediated EMT promoting effect on lung cancer cells. Cancer Lett..

[162] Jothimani G., Pathak S., Dutta S., Duttaroy A.K., Banerjee A. (2022). A Comprehensive Cancer-Associated MicroRNA Expression Profiling and Proteomic Analysis of Human Umbilical Cord Mesenchymal Stem Cell-Derived Exosomes. Tissue Eng. Regen. Med..

[163] Oppliger B., Joerger-Messerli M., Mueller M., Reinhart U., Schneider P., Surbek D.V., Schoeberlein A. (2016). Intranasal Delivery of Umbilical Cord-Derived Mesenchymal Stem Cells Preserves Myelination in Perinatal Brain Damage. Stem Cells Dev..

[164] Bodart-Santos V., de Carvalho L., de Godoy M.A., Batista A.F., Saraiva L.M., Lima L.G., Abreu C.A., De Felice F.G., Galina A., Mendez-Otero R. (2019). Extracellular vesicles derived from human Wharton’s jelly mesenchymal stem cells protect hippocampal neurons from oxidative stress and synapse damage induced by amyloid-β oligomers. Stem Cell Res. Ther..

[165] Gu D., Zou X., Ju G., Zhang G., Bao E., Zhu Y. (2016). Mesenchymal Stromal Cells Derived Extracellular Vesicles Ameliorate Acute Renal Ischemia Reperfusion Injury by Inhibition of Mitochondrial Fission through miR-30. Stem Cells Int..

[166] Zhang Y.Z., Liu F., Song C.G., Cao X.L., Zhang Y.F., Wu H.N., Guo C.J., Li Y.Q., Zheng Q.J., Zheng M.H. (2018). Exosomes derived from human umbilical vein endothelial cells promote neural stem cell expansion while maintain their stemness in culture. Biochem. Biophys. Res. Commun..

[167] Zhong Y., Luo L. (2021). Exosomes from Human Umbilical Vein Endothelial Cells Ameliorate Ischemic Injuries by Suppressing the RNA Component of Mitochondrial RNA-processing Endoribonuclease via the Induction of miR-206/miR-1-3p Levels. Neuroscience.

[168] Ilancheran S., Moodley Y., Manuelpillai U. (2009). Human fetal membranes: A source of stem cells for tissue regeneration and repair?. Placenta.

[169] Abumaree M.H., Abomaray F.M., Alshehri N.A., Almutairi A., AlAskar A.S., Kalionis B., Al Jumah M.A. (2016). Phenotypic and Functional Characterization of Mesenchymal Stem/Multipotent Stromal Cells from Decidua Parietalis of Human Term Placenta. Reprod. Sci..

[170] Miao Z., Jin J., Chen L., Zhu J., Huang W., Zhao J., Qian H., Zhang X. (2006). Isolation of mesenchymal stem cells from human placenta: Comparison with human bone marrow mesenchymal stem cells. Cell Biol. Int..

[171] Nekanti U., Mohanty L., Venugopal P., Balasubramanian S., Totey S., Ta M. (2010). Optimization and scale-up of Wharton’s jelly-derived mesenchymal stem cells for clinical applications. Stem Cell Res..

[172] Gupta D., Zickler A.M., El Andaloussi S. (2021). Dosing extracellular vesicles. Adv. Drug Deliv. Rev..

[173] Murali V.P., Holmes C.A. (2021). Biomaterial-based extracellular vesicle delivery for therapeutic applications. Acta Biomater..

[174] Pinheiro A., Silva A.M., Teixeira J.H., Gonçalves R.M., Almeida M.I., Barbosa M.A., Santos S.G. (2018). Extracellular vesicles: Intelligent delivery strategies for therapeutic applications. J. Control Release.

[175] Yan L., Wu X. (2020). Exosomes produced from 3D cultures of umbilical cord mesenchymal stem cells in a hollow-fiber bioreactor show improved osteochondral regeneration activity. Cell Biol. Toxicol..

[176] Xin L., Lin X., Pan Y., Zheng X., Shi L., Zhang Y., Ma L., Gao C., Zhang S. (2019). A collagen scaffold loaded with human umbilical cord-derived mesenchymal stem cells facilitates endometrial regeneration and restores fertility. Acta Biomater..

[177] Yang J., Chen Z., Pan D., Li H., Shen J. (2020). Umbilical Cord-Derived Mesenchymal Stem Cell-Derived Exosomes Combined Pluronic F127 Hydrogel Promote Chronic Diabetic Wound Healing and Complete Skin Regeneration. Int. J. Nanomed..

[178] Han C., Zhou J., Liang C., Liu B., Pan X., Zhang Y., Wang Y., Yan B., Xie W., Liu F. (2019). Human umbilical cord mesenchymal stem cell derived exosomes encapsulated in functional peptide hydrogels promote cardiac repair. Biomater. Sci..

